# Polysomnographic Parameters in Schizoaffective Disorder: A Systematic Review and Meta-Analysis

**DOI:** 10.31083/AP52900

**Published:** 2026-08-19

**Authors:** Dario Morra, Gianluca Ficca, Giuseppe Barbato

**Affiliations:** ^1^Department of Psychology, Università degli Studi della Campania “Luigi Vanvitelli”, 81100 Caserta, Italy

**Keywords:** REM, NREM, sleep, schizoaffective disorder, polysomnography, electroencephalography, meta-analysis

## Abstract

**Background:**

Schizoaffective disorder (SZA) is a severe psychiatric condition characterized by overlapping affective and psychotic features; however, its sleep architecture remains poorly characterized. This systematic review and meta-analysis compared polysomnographic findings in drug-free patients with SZA with those of healthy controls (HC), patients with schizophrenia (SCZ), and patients with major depressive disorder (MDD).

**Methods:**

An extensive database search identified 40 studies. Nine case-control studies including 79 patients with SZA, 88 patients with SCZ, 79 HC, and 131 patients with MDD were included in the meta-analyses. Total sleep time, sleep efficiency, sleep latency, wake after sleep onset, and rapid-eye-movement (REM) and non-REM (NREM) sleep variables were analyzed. The primary outcome measure was the standardized mean difference (SMD). Data were analyzed using a random-effects model. Publication bias was assessed using Egger’s regression test and funnel plot asymmetry.

**Results:**

Compared with HC, patients with SZA showed reduced total sleep time, increased sleep latency and wakefulness after sleep onset, reduced REM sleep duration, shortened REM latency, and reduced stage 4 sleep duration and percentage. Patients with SZA differed from those with MDD only in exhibiting increased sleep latency, whereas no significant differences were observed between patients with SZA and those with SCZ.

**Conclusions:**

Polysomnographic abnormalities in SZA demonstrate a general pattern of sleep disruption compared with HC, without clear differentiation from SCZ or MDD. Overall, current evidence does not support sleep architecture as a reliable biomarker for distinguishing SZA from related psychotic or affective disorders. These findings should be considered exploratory, given the small sample sizes, limited number of available studies, and substantial heterogeneity across studies. Further research is needed to determine whether specific sleep measures may better distinguish SZA from other psychiatric conditions.

## Main Point

1. Sleep parameters do not differentiate schizoaffective disorder (SZA) from major depressive disorder (MDD) and schizophrenia (SCZ).

2. SZA is associated with impaired sleep continuity compared with healthy controls (HC).

3. Delta sleep reduction appears across SZA, SCZ, and MDD compared with HC.

4. Polysomnographic evidence in SZA is limited, with small and heterogeneous samples.

## 1. Introduction

### 1.1 Schizoaffective Disorder

Schizoaffective disorder (SZA) is a chronic condition which, according to current DSM-5 criteria, can be diagnosed when a mood disorder (depression, mania or both) is present for the majority of the total active and residual course of schizophrenia (SCZ), from the onset of psychotic symptoms up until the current diagnosis [1,2,3]. SZA is estimated to have a lifetime prevalence of 0.3%–1.1% [1,4,5]. Age of onset of SZA is 25–35, intermediate between that of SCZ and mood disorders [6,7,8], although it might be broader [9]. Women are twice as likely to be diagnosed with SZA than men, as for mood disorders [7,8,10,11,12,13]. Mixed states in SZA have been reported to be as frequent as mixed states in bipolar disorder (BD) and probably linked to a worse outcome [14]. Lifetime suicide risk in SZA is estimated to be 5%, although the presence of depressive symptoms might increase it [8,15]. SZA is mostly regarded as having a more favorable course than that of patients with SCZ but worse than that of patients with BD [8,14,16,17].

The historical perspective proposed from the end of the nineteenth century onward by Kraepelin [18] suggested that SCZ and BD were the two main diagnostic categories, with the psychoses associated with the former category having a poorer outcome than those associated with mood disorders [19]. The dichotomy proposed by Kraepelin, however, did not acknowledge those cases in which patients experience psychotic symptoms but also have periods of concomitant mood disorders. The term “schizoaffective psychosis” was first used by Jacob Kasanin in 1933 [20]; therefore the “schizoaffective subtype” of “schizophrenic reaction” is present in DSM since its first 1952 edition [2]. SZA started to be regarded as a separate diagnostic entity in DSM-III, while successive editions added subtypes (depressed, bipolar, mixed) and from DSM-5 recognized SZA as a lifetime diagnosis [2]. International Classification of Diseases (ICD)-11 differs from DSM by dropping the longitudinal approach to the diagnosis of SZA, considering it difficult to ascertain the accurate temporal relationship between psychotic and mood episodes, and proposing the coexistence yet independence of the two classes of symptoms as a main criterion [3].

SZA has been generally regarded as having poor diagnostic reliability [1,2,3,4,5,6,13,21], making SZA a potentially misdiagnosed condition [2,22,23,24]. The question of whether SZA is an actual separate clinical entity or a subtype of SCZ or a depressive disorder has been frequently discussed in literature, contributing to the controversial nosography of SZA [6,7,8,21,22,25,26,27,28,29,30,31,32].

Several studies assessing clinical and biological features have been conducted to characterize SZA. Cognitive [33,34,35] and gray matter abnormalities [36,37,38,39] have been found in SZA, often resembling more those in SCZ than those in BD [8,34], or being intermediate between SCZ and BD [40]. SZA patients do not show a different ventricle-to-brain ratio compared to SCZ or BD [25,41]. Recent studies on fMRI [42], functional connectivity [43] and immune system [44,45] found that SZA, SCZ and BD share substantial biological abnormalities while also exhibiting partially distinct profiles.

Heritability of SZA seems to be like that of SCZ and BD [46]. Some studies found that SZA is genetically linked to both SCZ and BD and might constitute an intermediate form of these two conditions [47,48,49], with evidence that genes involved in glutamatergic abnormalities might play a key role in the onset of SZA, SCZ and BD [50]. In a recent study on Swedish population, SZA has been found to carry high genetic risk for both SCZ and BD while being clearly separable from psychotic BD, suggesting that SZA might not be, from a genetic perspective, a subtype of SZ, BD or MD [51]. As already suggested in early studies [25], SZA might be an intermediate condition in a putative polygenic continuum model of psychoses in which patients with major psychoses share genetic influences [52,53,54].

SZA is mainly treated with antipsychotics as SCZ [1,24,31,55], with paliperidone being the only FDA specifically approved medication for SZA [56,57]. Patients with SZA, nonetheless, receive additional prescription of benzodiazepines, antidepressants and mood stabilizers [1,17,24] and with a higher frequency compared to SCZ [17]. Electroconvulsive therapy (ECT) is also effective in treating treatment-resistant SZA [8,24].

### 1.2 Sleep Studies of SZA

Insomnia [58,59,60], obstructive sleep apnea (or at risk for it) [59,61,62] and circadian rhythm disorders [59,63] are sleep disturbances reported in SZA. Insomnia has been found to be associated with obesity [58] and suicidal ideation and risk in SZA patients [64]. Sleep disturbances were also associated with more severe negative symptoms and lower functioning [60]. A study that used a sleep spindles detection method reported different spindle phenotypes between SZA and SCZ; moreover, both slow and fast sleep spindles density were increased in those patients with a history of manic or hypomanic symptoms [65]. A recent study reported reduced thalamic nuclei volumes in SZA compared with SCZ [66], a potentially relevant finding given the central involvement of thalamo-cortical circuits in sleep spindles generation [67]. Notably, spindle deficits have already been consistently documented in SCZ [68,69].

The sleep of patients with SZA was first investigated in early descriptive studies, reporting abnormalities in REM percentage (%REM) and in slow-wave sleep (SWS) [70]. Another study reported normal REM latency in SZA but increased REM density (REMD) in depressed SZA patients compared to manic BD patients, manic SZA patients and SCZ [71]. Several other studies pooled SZA patients in samples together with SCZ patients, limiting the possibility to observe differences within the sample in sleep parameters.

Nonetheless, sleep architecture in samples composed entirely of SZA patients was investigated in case-control studies comparing patients with healthy controls (HC). The earliest study reported increased sleep onset latency (SOL) and wake after sleep onset (WASO) in SZA, with stage 4 duration (ST4) and percentage (%ST4) and delta sleep time (DELTA) deficits [72]. Subsequent studies supported these findings by also reporting reduced total sleep time (TST) and REM time (REMT) and shortened REM latency (REML) in SZA [73,74,75].

Other studies investigated differences between the sleep architecture of SZA and that of SCZ or affective disorder patients. Reich et al. [76] reported increased SOL, decreased sleep efficiency (SE) and decreased DELTA in SZA compared to SCZ, with SZA also showing shortened REML and increased REMT and REMD. Subsequent studies reported increased ST4 [72,73], %ST4 and %REM in SCZ compared to SZA [73].

An early study found decreased WASO and increased REMT and DELTA in SZA patients compared to those with psychotic depression [77], although a later report by the same authors found no differences between SZA and psychotic depression [78].

Furthermore no differences between SZA, SCZ and MDD were reported by successive studies [73,74,75].

Considering the previous heterogeneous and controversial results, the present study aimed to provide a qualitative and quantitative analysis of PSG findings in SZA.

In addition to quantitative synthesis, the systematic review includes studies that could not be subjected to meta-analysis due to insufficient or incompatible data but providing a more comprehensive characterization of the available evidence. As a novel contribution, the study further enables an exploratory comparative assessment of sleep architecture alterations shared across major psychiatric conditions to clarify the degree of similarity of SZA sleep with that of SCZ or MDD. TST, SOL, SE, WASO, REMT, %REM, REML, REMD, stage 1, 2, 3 sleep time (ST1, ST2, ST3) and percentage (%ST1, %ST2, %ST3), ST4, %ST4, DELTA and %DELTA in drug-free SZA patients were analyzed and, where available, compared with case-control data of HC, MDD and SCZ patients.

This study is structured as follows: first, the methods used for the systematic review and meta-analysis are described, including study selection, polysomnographic variables, and statistical procedures. The results section then presents the meta-analytic findings. Finally, the discussion addresses the interpretation of the findings in relation to previous literature, the main limitations of the study, potential sources of heterogeneity, and implications for future research.

## 2. Methods

Major databases were screened querying PubMed, Web of Science and PsycINFO from inception to 2025. Several combinations of keywords were implemented to identify all the studies of interest: “(sleep or sleep*) AND schizo*”, “PSG or polysomnogra*”, “EEG OR electroencephalogra*”, “REM OR REMS OR rapid-eye-movement OR rapid-eye-movements”, “paradox OR paradox*”, “print OR print*”, “D-phase OR dream OR dream*”, “night OR night*”, “monit*”, “architect*”. In addition, keywords regarding molecules and brand names were included to identify studies investigating the effect of psychotropic drugs on sleep architecture, such as: “atypical”, “typical” and “antipsych*”, “paliperidone”, “risperidone”, “Invega”, “Risperdal” and so on. Although drug-related keywords were included in the search strategy to maximise sensitivity and capture PSG outcomes reported within pharmacological studies, no eligible studies investigating drug effects on sleep in SZA were ultimately identified or included in the final dataset.

The search returned 7515 studies, of which 3345 from PubMed, 4105 from WoS and 65 from PsycINFO. Of these, 4120 studies were found to be duplicates present on multiple databases. The eligibility of the remaining 3395 studies was assessed, with 3202 studies eventually excluded due to not providing any polysomnography data. 13 studies were excluded due to providing patient samples already presented in a previous study by the same author or co-author. 144 studies were excluded due to not having any SZA patients in their sample. 36 studies were evaluated as eligible and entirely retrieved. 40 studies were ultimately included in the study after being assessed as eligible: 36 studies were retrieved from database screening and 4 other studies were identified through manual search and retrieved. The meta-analysis included 9 studies, while the systematic review included all the other 31 studies. The eligibility assessment of each study was carried out after a full-text review of the articles by the authors (DM, GF and GB), all agreeing on the final decision for each study.

**Fig. 1.**
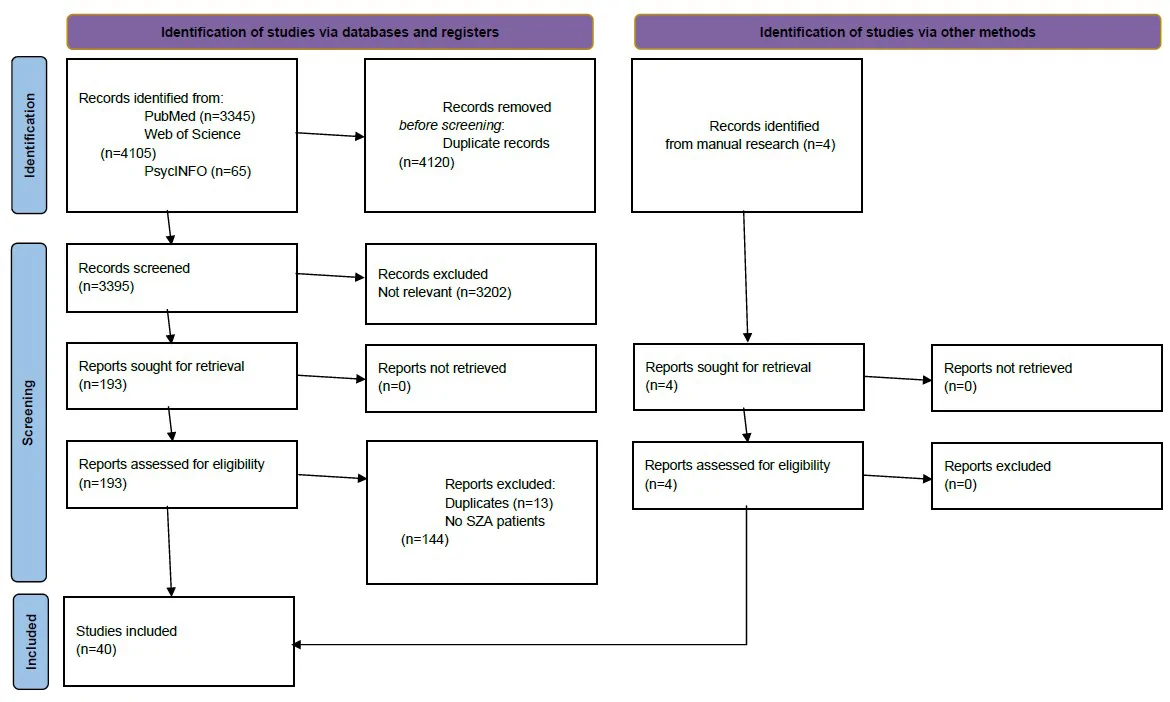
**PRISMA diagram**.

The meta-analysis investigated sleep architecture differences between the following groups: drug-free SZA and HC (Table 1, Ref. [72,73,74,75]), drug-free SZA and drug-free MDD (Table 2, Ref. [72,73,74,75,77,78,79,80]), drug-free SZA and drug-free SCZ (Table 3, Ref. [72,73,74,75,76,80]). Case-control studies that were eligible for the systematic review only (and excluded from the meta-analysis) are reported in Table 4 (Ref. [65,81,82,83,84,85,86,87,88,89,90,91,92,93,94]) with the reason for exclusion. Other non-case-control studies included in the systematic review only are reported in Table 5 (Ref. [70,71,95,96,97,98,99,100,101,102,103,104,105,106,107,108]). Fig. 1 presents the PRISMA (Preferred reporting items for systematic reviews and meta-analyses) diagram of the systematic review.

**Table 1.** **Drug-free SZA vs HC studies included in the meta-analysis**.

Study	N. (SZA)/N. (HC)	Age (SZA/HC)	Males % (SZA/HC)	Diagnosis (SZA/HC)	Treatment	Diagnostic manual
Benson et al., 1980 [72]	9/19	NR/NR	NR/NR	Schizoaffective disorder/Healthy	Drug-free (≥2 weeks)	Research Diagnostic Criteria
Benson & Zarcone, 1985 [73]	8/13	33/29.7	100/100	Schizoaffective disorder/Healthy	Drug-free (≥2 weeks)	Research Diagnostic Criteria
Benson & Zarcone, 1985 [73]	9/19	33.4/30.2	100/100	Schizoaffective disorder/Healthy	Drug-free (≥2 weeks)	Research Diagnostic Criteria
Zarcone et al., 1987 [74]	8/18	33.8/30.3	100/100	Schizoaffective disorder/Healthy	Drug-free (≥2 weeks)	Research Diagnostic Criteria
Zarcone & Benson, 1995 [75]	8/10	34.5/32.3	100/100	Schizoaffective disorder/Healthy	Drug-free (2 days)	Research Diagnostic Criteria

“NR”: data not reported in the original study. SZA, schizoaffective disorder; HC, healthy control.

**Table 2.** **Drug-free SZA vs drug-free MDD studies included in the meta-analysis**.

Study	N. (SZA)/N. (MDD)	Age (SZA/MDD)	Males % (SZA/MDD)	Diagnosis (SZA/MDD)	Treatment	Diagnostic manual
Kupfer & Foster, 1975 [77]	6/9	38.8/58.2	50/NR	Schizoaffective disorder/Psychotic depression	Drug-free (≥2 weeks)	Research Diagnostic Criteria
Kupfer & Foster, 1975 [77]	6/25	38.8/41.1	50/NR	Schizoaffective disorder/MDD	Drug-free (≥2 weeks)	Research Diagnostic Criteria
Kupfer et al., 1979 [78]	12/29	44.4/48.1	42/31	Schizoaffective disorder/Psychotic depression	Drug-free (≥2 weeks)	Research Diagnostic Criteria
Benson et al., 1980 [72]	9/6	NR/NR	NR/NR	Schizoaffective disorder/MDD	Drug-free (≥2 weeks)	Research Diagnostic Criteria
Coble et al., 1981 [79]	5/17	34.8/40.1	60/35	Schizoaffective disorder/Psychotic and nonpsychotic depression	Drug-free (2 weeks)	Research Diagnostic Criteria
Benson et al., 1983 [80]	2/5	NR/NR	100/100	Schizoaffective disorder/MDD	Drug-free (≥2 weeks)	Research Diagnostic Criteria
Benson & Zarcone, 1985 [73]	8/10	33/40.3	100/100	Schizoaffective disorder/MDD	Drug-free (≥2 weeks)	Research Diagnostic Criteria
Benson & Zarcone, 1985 [73]	9/7	33.4/37.7	100/100	Schizoaffective disorder/MDD	Drug-free (≥2 weeks)	Research Diagnostic Criteria
Zarcone et al., 1987 [74]	8/12	33.8/41.33	100/100	Schizoaffective disorder/MDD	Drug-free (≥2 weeks)	Research Diagnostic Criteria
Zarcone & Benson, 1995 [75]	8/11	34.5/37.2	100/100	Schizoaffective disorder/MDD	Drug-free (2 days)	Research Diagnostic Criteria

“NR”: data not reported in the original study. MDD, major depressive disorder.

**Table 3.** **Drug-free SZA vs drug-free SCZ studies included in the meta-analysis**.

Study	N. (SZA)/N. (SCZ)	Age (SZA/SCZ)	Males % (SZA/SCZ)	Diagnosis (SZA/SCZ)	Treatment	Diagnostic manual
Reich et al., 1975 [76]	3/14	38.8/20.8	NR/57	Schizoaffective disorder/Schizophrenia (acute)	Drug-free (≥2 weeks)	DSM-II
Reich et al., 1975 [76]	3/9	38.8/19	NR/11	Schizoaffective disorder/Schizophrenia (latent)	Drug-free (≥2 weeks)	DSM-II
Benson et al., 1980 [72]	9/9	NR/NR	NR/NR	Schizoaffective disorder/Schizophrenia	Drug-free (≥2 weeks)	Research Diagnostic Criteria
Benson et al., 1983 [80]	2/7	NR/NR	100/100	Schizoaffective disorder/Schizophrenia	Drug-free (≥2 weeks)	Research Diagnostic Criteria
Benson & Zarcone, 1985 [73]	8/11	33/27	100/100	Schizoaffective disorder/Schizophrenia	Drug-free (≥2 weeks)	Research Diagnostic Criteria
Benson & Zarcone, 1985 [73]	9/8	33.4/26.6	100/100	Schizoaffective disorder/Schizophrenia	Drug-free (≥2 weeks)	Research Diagnostic Criteria
Zarcone et al., 1987 [74]	8/12	33.8/26.2	100/100	Schizoaffective disorder/Schizophrenia	Drug-free (≥2 weeks)	Research Diagnostic Criteria
Zarcone & Benson, 1995 [75]	8/18	34.5/33.1	100/100	Schizoaffective disorder/Schizophrenia	Drug-free (2 days)	Research Diagnostic Criteria

“NR”: data not reported in the original study. SCZ, schizophrenia.

**Table 4.** **Case-control studies included in the systematic review only**.

Study (excluded)	N. (SZA)/N. (CTRL)	Age (SZA/CTRL)	Males % (SZA/CTRL)	Diagnosis (SZA/CTRL)	Treatment	Reason of exclusion	Diagnostic manual
Kupfer et al., 1971 [81]	4	36.48	25	1 Schizophrenia 1 Schizoaffective disorder 2 Depressive neurosis	Drug-free (≥2 weeks) → Chlorpromazine	Mixed sample	Not reported
Gillin et al., 1977 [82]	10	25	60	4 Schizoaffective disorder 2 Acute schizophrenia 1 Chronic schizophrenia 1 Schizoid personality disorder 2 Bipolar disorder	Drug-free (≥2 weeks) → Pimozide	Mixed sample	DSM-II
Gillin et al., 1978 [83]	7	23	43	3 Chronic schizophrenia 1 Acute schizophrenia 2 Schizoaffective disorder 1 Schizoid personality disorder + OCD	Drug-free (≥1 month) → Pimozide	Mixed sample	Research Diagnostic Criteria
Keshavan et al., 1996 [84]	16	29.9	50	11 Schizophrenia 5 Schizoaffective disorder	Drug-free (≥2 weeks) → Drug-free (31%) Neuroleptics (69%) + Antidepressants + Mood stabilizers	Mixed medication status/sample	DSM-III-R
Keshavan et al., 1996 [84]	15	29.7	NR	Schizophrenia 5 Schizoaffective disorder	Drug-free (≥2 weeks) → Haloperidol (67%) Fluphenazine (13%) Trifluoperazine (6.7%) Clozapine (6.7%) Risperidone (6.7%) + Anticholinergics (47%)	Mixed sample	DSM-III-R
Benson et al., 1996 [85]	14/15	33.6/30.5	100/100	11 Schizophrenia (presumably chronic) 3 Schizoaffective disorder/Healthy	Drug-free (2 days)	Mixed sample	Research Diagnostic Criteria
Nishino et al., 1998 [86]	14/14	33.3/34.4	100/100	12 Schizophrenia 2 Schizoaffective disorder/Healthy	Drug-free (2 days)	Mixed sample	Research Diagnostic Criteria
Keshavan et al., 1998 [87]	30/30	30.9/30.9	57/57	19 Schizophrenia 8 Schizoaffective disorder 3 Schizophreniform disorder/Healthy	Drug-free (Average 97.5 weeks; Median 40 weeks)	Mixed sample	DSM-III-R
Rotenberg et al., 2000 [88]	20/10	45.8/43.3	65/60	19 Schizophrenia 1 Schizoaffective disorder/Healthy	Drug-free (≥2 weeks)	Mixed sample	DSM-IV
Armitage et al., 2004 [89]	14	40.9	29	3 Schizoaffective disorder 11 Bipolar I disorder	Carbamazepine, BZD, mood stabilizers → + Clozapine	Mixed sample	DSM-IV
Kluge et al., 2014 [90]	15	32.8	47	Schizophrenia 1 Schizoaffective disorder	Drug-free (≥2 days) → Olanzapine	Mixed sample	DSM-IV
Kluge et al., 2014 [90]	15	36.7	33	Schizophrenia 2 Schizoaffective disorder 1 Schizophreniform disorder	Drug-free (≥2 days) → Clozapine	Mixed sample	DSM-IV
Göder et al., 2015 [91]	16/16	29.4/28.3	56/44	14 Chronic schizophrenia 2 Schizoaffective disorder/Healthy	Antipsychotics (2° Gen., 94%) Antipsychotics (1° Gen., 6%) + Antidepressants (13%) + BZD (6%) + Anticholinergics (6%) + Promethazine (6%)	Mixed sample	ICD-10
Kaskie et al., 2019 [92]	27/23	23.2/24.7	68/72	16 Schizophrenia 2 Schizoaffective disorder 4 Unspecified psychosis 3 MDD with psychotic features 2 Bipolar disorder/Healthy	Antipsychotics (2° Gen., 41%) Antipsychotics (3° Gen., 3.7%) + Antidepressants (83%) + Mood stabilizers (25%) + BZD (17%) 15 drug-naive	Mixed medication status/sample	DSM-IV
Gerstenberg et al., 2020 [93]	12/24	16.6/16.5	58/46	9 Schizophrenia 2 Schizoaffective Disorder 1 Brief Psychotic Disorder/Healthy	Antipsychotics (3° Gen., 58.3%) Antipsychotics (2°+3° Gen., 25.5%) Antipsychotics (2° Gen., 16.2%) + BZD (25%) + Anticholinergics (16.7%)	Mixed sample	DSM-IV
Petit et al., 2022 [65]	12/988	58.2/59.9	42/58	Schizoaffective disorder/Healthy	Medicated (25%) Drug-free (75%)	Mixed medication status	DSM-5
Denis et al., 2024 [94]	17/27	21/24	56/53	7 Schizophrenia 5 Schizoaffective disorder 4 Bipolar disorder 1 Unspecified psychosis/Healthy	Antipsychotics (2° Gen., 35%) Antipsychotics (3° Gen., 29%) Antipsychotics (1° Gen., 12%) + Mood stabilizers (47%) + Antidepressants (24%) + Anticholinergics (18%) + BZD (6%) 4 drug-free	Mixed medication status/sample	DSM-IV-TR

CTRL, Control group; DSM, Diagnostic and Statistical Manual of Mental Disorders; OCD, Obsessive-Compulsive Disorder; BZD, Benzodiazepines; ICD, International Classification of Diseases. “NR”: data not reported in the original study.

**Table 5.** **Non-case-control studies included in the systematic review only**.

Study	N. SZA/Sex/(Age)	Diagnosis	Medication status	Diagnostic manual
Hauri et al., 1973 [70]	2F (28.5)	Schizoaffective disorder	Drug-free (≥1 week)	Not reported
Gillin et al., 1981 [95]	3M, 3F (31)	4 Chronic schizophrenia 2 Schizoaffective disorder	Drug-free (≥1 week)	Research Diagnostic Criteria
Glassman et al., 1986 [96]	1M (39)	Schizoaffective disorder	Lithium carbonate, chlorpromazine, triazolam, benztropine	DSM-III
Lahmeyer et al., 1988 [71]	2F (29)	Schizoaffective disorder	Drug-free (2 weeks)	Research Diagnostic Criteria
Keshavan et al., 1991 [97]	7M, 5F (31.2)	8 Schizophrenia 3 Schizoaffective disorder 1 Schizotypal disorder	Drug-free (≥2 weeks) 2 drug-naive	Research Diagnostic Criteria
Keshavan et al., 1992 [98]	14M, 5F (30.5)	17 Schizophrenia 2 Schizoaffective disorder	Drug-free (≥2 weeks) 6 drug-naive	Research Diagnostic Criteria
Douglass et al., 1993 [99]	6NR (NR)	Schizoaffective disorder Schizophrenia	Drug-free (≥2 weeks)	DSM-III-R
Douglass et al., 1993 [99]	3NR (NR)	Schizophrenia Schizoaffective disorder + narcolepsy	Drug-free (≥2 weeks)	DSM-III-R
Keshavan et al., 1994 [100]	8M, 11F (30.6)	13 Schizophrenia 6 Schizoaffective disorder (suicidal behavior)	Drug-free (≥2 weeks)	DSM-III-R
Keshavan et al., 1994 [100]	12M, 8F (32.3)	16 Schizophrenia 4 Schizoaffective disorder (no suicidal behavior)	Drug-free (≥2 weeks)	DSM-III-R
Keshavan et al., 1995 [101]	10M, 10F (30)	8 Schizoaffective disorder 12 Schizophrenia	Drug-free (≥4 weeks) 12 drug-naive	DSM-III-R
Yamashita et al., 1999 [103]	1F (34)	Schizoaffective disorder (with haloperidol-induced akathisia)	Haloperidol + Chlorpromazine + Biperiden + BZD + Mood stabilizer → BZD + Mood stabilizer	DSM-IV
Wetter et al., 2002 [104]	1F (31)	Schizoaffective disorder (with risperidone-induced restless leg syndrome)	Risperidone + Mood stabilizers → Quetiapine + Mood stabilizers	NR
Baandrup et al., 2016 [102]	5M, 5F (49)	6 Schizophrenia 2 Schizoaffective disorder 2 Bipolar disorder	Antipsychotics + BZD + Antidepressants + Mood stabilizers + Anticholinergics	ICD-10
Weinhold et al., 2022 [105]	9M, 9F (40.7)	11 Schizophrenia 7 Schizoaffective disorder	Atypical antipsychotics + Antidepressants + Mood stabilizers	ICD-10
			**Study summary (no clear sleep data)**	
Itil et al., 1970 [108]	1NR (NR)	Schizoaffective disorder	Drug-free (≥4 weeks)	Not reported
Itil et al., 1970 [108]	1NR (NR)	Schizoaffective disorder (lobotomized)	Drug-free (≥4 weeks)	Not reported
Giedke et al., 1992 [106]	12M, 18F (48)	4 Schizoaffective disorder 26 MDD	All patients on neuroleptics, antidepressants and BZD Sleep deprivation significantly improved depressed mood	Research Diagnostic Criteria
Keshavan et al., 1992 [107]	11M, 5F (31.65)	10 Schizophrenia 4 Schizoaffective disorder 1 Atypical psychosis 1 Psychotic affective disorder	Reduced NREM sleep REM activity increased, REML reduced (drug-free ≥2 days) Neuroleptic treatment did not improve sleep	DSM-III-R

“NR”: data not reported in the original study.

### 2.1 Eligibility Criteria

Studies were assessed as eligible for the meta-analysis based on the following inclusion criteria:

(1) Case-control studies including a sample of patients with SZA and a HC group.

(2) Case-control studies including a sample of patients with SZA and a MDD sample.

(3) Case-control studies including a sample of patients with SZA and a SCZ sample.

(4) Patient samples in a study had been drug-free for the same amount of time.

(5) Samples aged over 13 years.

(6) Studies in which diagnoses were established according to DSM and/or ICD criteria. Research diagnostic criteria (RDC) have been widely used to diagnose SZA and were therefore regarded as eligible.

(7) Studies in which sleep was assessed by polysomnography (PSG) and sleep stages were scored according to AASM or Rechtschaffen and Kales criteria. Studies using earlier scoring systems (e.g., Antrobus et al. [109], or Itil et al. [110]) were also included provided that the scoring methods were equivalent or largely comparable to those adopted in the later standard guidelines.

Exclusion from the meta-analysis was based on the following criteria:

(1) Studies with no control group.

(2) Studies with a control group different than SZA, SCZ, MDD or HC.

(3) Studies in which more than 5% of the total sample consisted of participants with diagnoses other than SZA (e.g., obsessive-compulsive disorder, bipolar disorder, or autism spectrum disorder).

(4) Studies in which the SZA sample included a subgroup with a different medication status from the majority of participants, exceeding 5% of the total sample (e.g., 90% drug-free and 10% receiving at least one antipsychotic).

(5) Studies including SZA patients with relevant comorbidities (e.g., narcolepsy or obstructive sleep apnea-hypopnea)

(6) Single-case studies.

(7) Studies reporting sleep alterations in SCZ patients without providing clearly extractable sleep data.

The excluded studies were retained in the systematic review.

As specified, studies were excluded if the SZA sample was not sufficiently homogeneous with respect to diagnostic composition or medication status. A conservative threshold (>5% of the sample) was applied [111] to minimize potential confounding arising from the inclusion of participants with different primary diagnoses and from psychotropic medication exposure, both of which may significantly influence sleep architecture and PSG parameters. The 5% cut-off is inherently arbitrary and is acknowledged as a methodological limitation of the present work.

The quality of each study included in either the systematic review or the meta-analysis was assessed using the Newcastle-Ottawa Scale. Item-level scores for each included study are provided as supplementary material.

### 2.2 Data Extraction

The following data were extracted from each study: sample size, mean age, percentage of male participants, diagnosis, medication status, and mean and SD of sleep variables, including TST, SOL, SE, WASO, REMT, %REM, REML, REMD, ST1, ST2, ST3, ST4, %ST1, %ST2, %ST3, %ST4, DELTA, and %DELTA. In some studies, SDs for sleep variables were not reported. In these cases, SDs were estimated as follows. For each meta-analysis (e.g., REMT, SZA vs HC), a weighted average SD was computed based on the sample size of the included studies. This weighted SD was then assigned to studies within the same meta-analysis that did not report SDs. This approach has been considered acceptable for handling missing variance data [112] and represents the only imputation procedure applied in this study. In a limited number of studies, some sleep variables were not directly reported but could be derived from other available parameters (e.g., REMT = TST × %REM / 100). All instances of data derivation and imputation were reported in the **Supplementary Material**, where the meta-analytic and systematic review data are presented in extended form.

Eight of the nine studies included in the meta-analysis used the RDC for diagnosing SZA, while only one study applied DSM-II criteria. The diagnostic system used in each study, including those excluded from meta-analysis, is reported in Tables 1,2,3,4.

In several included studies, demographic variables such as age or sex were not reported. This is indicated in Tables 1,2,3,4 with a “NR” denoting unavailable data.

Some studies were reported multiple times in the corresponding tables for they included multiple independent patient samples or diagnostic subgroups that constituted separate datasets to the analyses. Therefore, repeated citations do not represent duplicate inclusion of the same sample, but distinct patient samples analyzed separately within the original study.

### 2.3 Statistical Analysis

Jamovi 2.6.44 (Windows x64 desktop version, Sydney, Australia) was used to perform the meta-analyses. R 4.6.0 (Windows x64 desktop version, Auckland, New Zealand), together with the metaPower 0.2.2 package (Houston, Texas), was used to compute statistical power for each meta-analysis. A minimum of three datasets from independent studies was required for each parameter to conduct a meta-analysis. Analyses were performed using standardized mean differences as the effect size metric.

A random-effects model was fitted to the data. Between-study heterogeneity (τ^2^) was estimated using the restricted maximum-likelihood estimator. In addition to τ^2^, the Q-test for heterogeneity and the I^2^ statistic are reported. When any heterogeneity was detected (τ^2^ >0, irrespective of the Q-test outcome), a prediction interval for the true effect sizes was also calculated. Studentized residuals and Cook’s distances were used to identify potential outliers and influential studies within each model. Studies with studentized residuals exceeding the 100 × (1 − 0.05/(2k))th percentile of a standard normal distribution were considered potential outliers, corresponding to a Bonferroni-adjusted two-sided α = 0.05 for k studies. Studies with Cook’s distances greater than the median plus six times the interquartile range were considered influential. Publication bias and small-study effects were assessed using rank correlation test and regression test, with the standard error of the observed effect sizes as predictor of asymmetry.

## 3. Results

### 3.1 Study Characteristics

Nine studies, comprising 79 SZA patients, 88 SCZ patients, 79 HC and 131 MDD patients, were included in the meta-analysis [72,73,74,75,76,77,78,79,80]. Some studies contributed with more than one dataset to each sleep parameter analysis.

Additional 31 studies, including 113 SZA patients, 263 SCZ patients, 1031 HC and 31 MDD patients, were included in the systematic review [65,70,71,81,82,83,84,85,86,87,88,89,90,91,92,93,94,95,96,97,98,99,100,101,102,103,104,105,106,107,108].

**Fig. 2.**
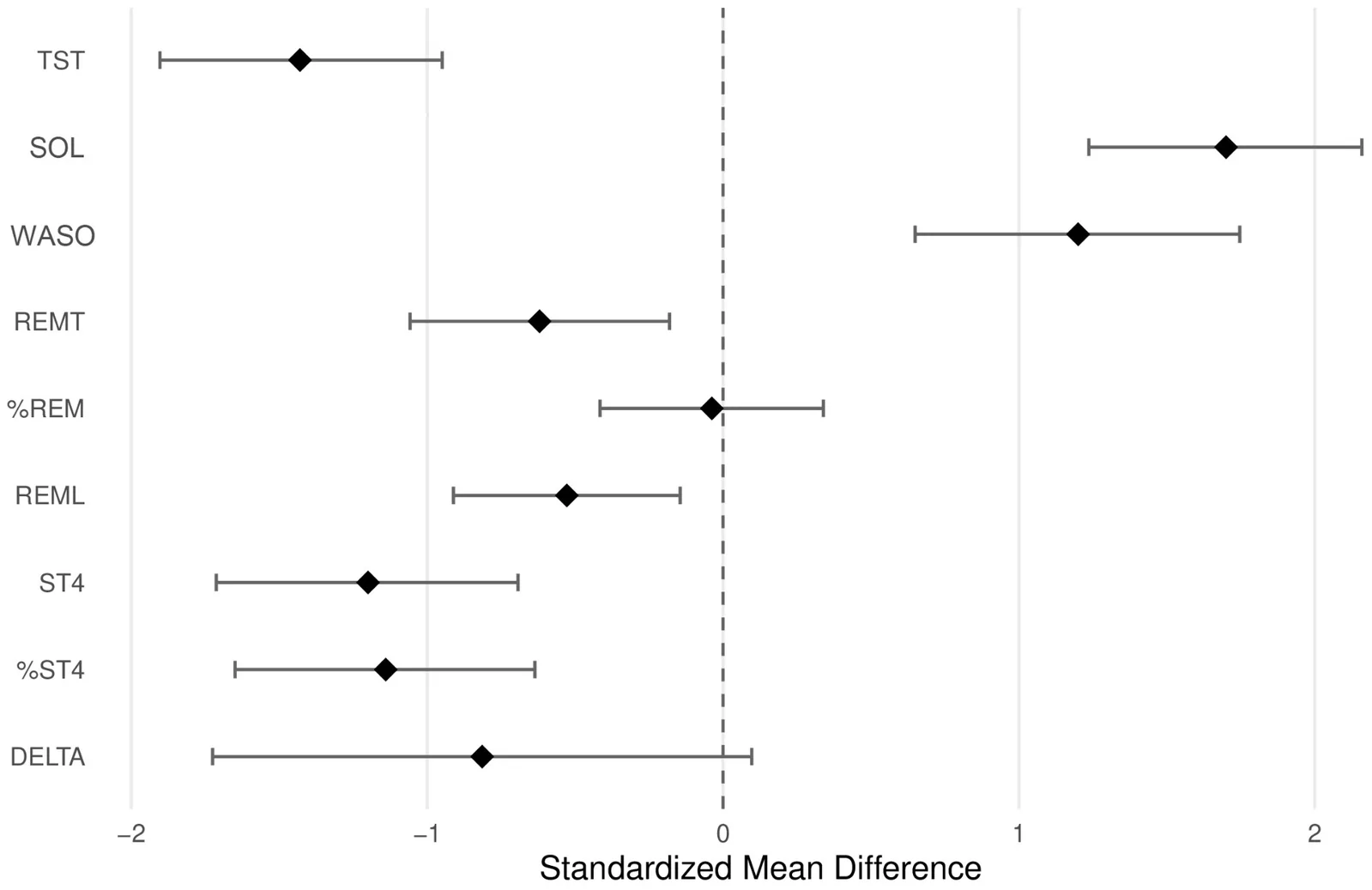
**Sleep parameters in drug-free SZA vs HC (forest plot)**.

**Fig. 3.**
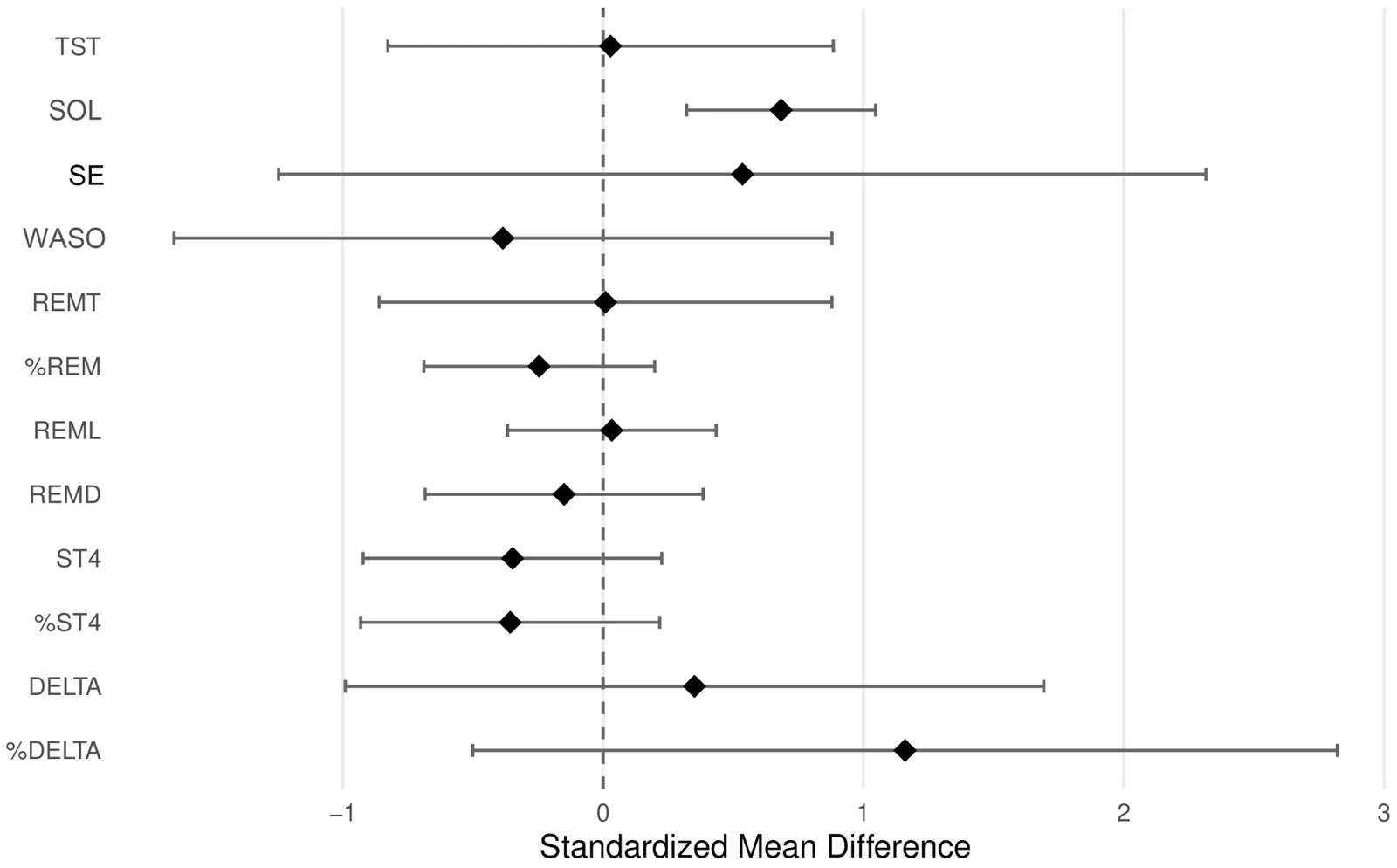
**Sleep parameters in drug-free SZA vs drug-free MDD (forest plot)**.

**Fig. 4.**
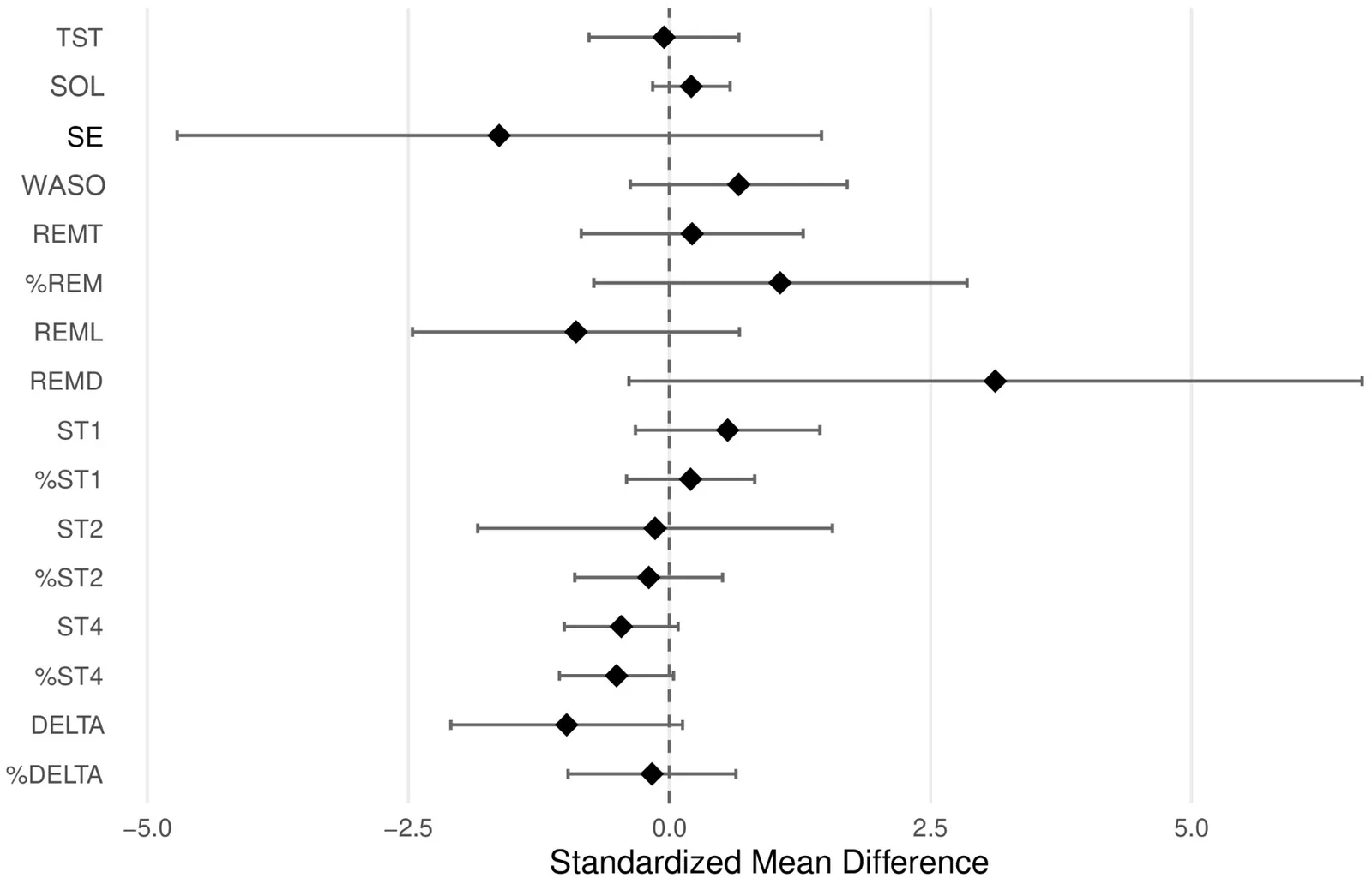
**Sleep parameters in drug-free SZA vs drug-free SCZ (forest plot)**.

Results are summarised in Table 6. Publication bias and funnel plot asymmetry, assessed using the rank correlation and regression tests, are reported in Table 7 (Ref. [72,74,75,76,77,80]), together with information on outliers and overly influential studies. Results from the meta-analyses comparing drug-free SZA with HC, drug-free MDD and drug-free SCZ are presented in forest plots in Figs. 2,3,4, respectively.

**Table 6.** **Meta-analysis results**.

	Meta-analysis				
SZA vs HC	N. of datasets	N. of subjects	Mean ± SD	RE Model	*p*-value
TST	4	33 vs 60	371.29 ± 72.7 vs 443.29 ± 28.72	–1.43 (–1.903 to –0.949)	***p***** < 0.001**
SOL	5	42 vs 79	51.78 ± 39.47 vs 11 ± 8.09	1.70 (1.236 to 2.159)	***p***** < 0.001**
WASO	3	25 vs 47	46.17 ± 42.34 vs 12.83 ± 10.94	1.20 (0.649 to 1.746)	***p***** < 0.001**
REMT	4	33 vs 60	79.7 ± 29.22 vs 96.85 ± 20.05	–0.620 (–1.058 to –0.181)	***p***** = 0.006**
%REM	5	42 vs 79	21.65 ± 5.98 vs 22.3 ± 5.02	–0.038 (–0.416 to 0.339)	*p* = 0.843
REML	5	42 vs 60	66.35 ± 28.33 vs 86.26 ± 34.76	–0.528 (–0.911 to –0.145)	***p***** = 0.007**
ST4	3	26 vs 51	7.65 ± 16.17 vs 47.41 ± 37.00	–1.20 (–1.713 to –0.693)	***p***** < 0.001**
%ST4	3	26 vs 51	1.88 ± 4.63 vs 10.53 ± 8.30	–1.14 (–1.649 to –0.636)	***p***** < 0.001**
DELTA	3	25 vs 47	43.16 ± 32.25 vs 74.56 ± 30.90	–0.814 (–1.725 to 0.097)	*p* = 0.080
	**Meta-analysis**				
**SZA vs MDD**	**N. of datasets**	**N. of subjects**	**Mean ± SD**	**RE Model**	***p*****-value**
TST	7	53 vs 83	345.56 ± 67.65 vs 344.43 ± 58.48	0.028 (–0.827 to 0.884)	*p* = 0.948
SOL	7	56 vs 80	57.82 ± 43.48 vs 37.68 ± 33.80	0.684 (0.321 to 1.047)	***p***** < 0.001**
SE	3	26 vs 49	68.52 ± 15.42 vs 67.19 ± 17.64	0.535 (–1.247 to 2.316)	*p* = 0.556
WASO	5	43 vs 67	40.08 ± 37.69 vs 42.41 ± 36.98	–0.385 (–1.649 to 0.879)	*p* = 0.550
REMT	7	53 vs 83	72.89 ± 25.6 vs 78.23 ± 35.85	0.009 (–0.861 to 0.879)	*p* = 0.984
%REM	8	62 vs 89	21.08 ± 5.65 vs 22.71 ± 6.71	–0.246 (–0.689 to 0.198)	*p* = 0.277
REML	10	67 vs 131	54.39 ± 25.57 vs 45.52 ± 22.90	0.033 (–0.367 to 0.434)	*p* = 0.870
REMD	3	20 vs 43	1.77 ± 0.71 vs 1.84 ± 0.95	–0.150 (–0.684 to 0.384)	*p* = 0.582
ST4	3	26 vs 23	7.65 ± 16.17 vs 14.78 ± 25.23	–0.348 (–0.922 to 0.225)	*p* = 0.234
%ST4	3	26 vs 23	1.88 ± 4.63 vs 3.60 ± 5.59	–0.357 (–0.932 to 0.217)	*p* = 0.223
DELTA	6	49 vs 92	26.74 ± 15.60 vs 21.26 ± 12.90	0.351 (–0.991 to 1.693)	*p* = 0.609
%DELTA	3	26 vs 49	5.42 ± 3.28 vs 2.22 ± 2.67	1.16 (–0.501 to 2.821)	*p* = 0.171
	**Meta-analysis**				
**SZA vs SCZ**	**N. of datasets**	**N. of subjects**	**Mean ± SD**	**RE Model**	***p*****-value**
TST	7	41 vs 79	368.62 ± 65.88 vs 369.82 ± 48.07	–0.052 (–0.770 to 0.666)	*p* = 0.887
SOL	6	44 vs 65	58.06 ± 36.12 vs 57.30 ± 36.78	0.210 (–0.160 to 0.581)	*p* = 0.266
SE	3	14 vs 41	71.80 ± 14.40 vs 78.63 ± 7.47	–1.63 (–4.714 to 1.458)	*p* = 0.301
WASO	5	31 vs 62	40.08 ± 32.34 vs 21.16 ± 14.97	0.664 (–0.375 to 1.703)	*p* = 0.210
REMT	7	41 vs 79	80.4 ± 25.6 vs 83.17 ± 18.53	0.218 (–0.845 to 1.281)	*p* = 0.688
%REM	8	50 vs 88	21.89 ± 5.36 vs 19.74 ± 2.86	1.06 (–0.724 to 2.850)	*p* = 0.244
REML	8	50 vs 88	62.27 ± 24.85 vs 67.15 ± 19.65	–0.894 (–2.461 to 0.672)	*p* = 0.263
REMD	3	8 vs 30	2.31 ± 0.20 vs 1.82 ± 0.10	3.12 (−0.388 to 6.635)	*p* = 0.081
ST1	3	14 vs 41	43.48 ± 8.9 vs 37.74 ± 4.69	0.558 (–0.326 to 1.441)	*p* = 0.216
%ST1	3	14 vs 41	12.63 ± 5.6 vs 10.76 ± 4.70	0.203 (–0.411 to 0.817)	*p* = 0.516
ST2	4	23 vs 50	194.74 ± 18.30 vs 201.45 ± 11.41	–0.137 (–1.836 to 1.561)	*p* = 0.874
%ST2	3	14 vs 41	56.02 ± 6.20 vs 58.83 ± 6.30	–0.198 (–0.907 to 0.510)	*p* = 0.583
ST4	3	26 vs 28	7.65 ± 16.17 vs 17.38 ± 26.08	–0.461 (–1.007 to 0.084)	*p* = 0.097
%ST4	3	26 vs 28	1.88 ± 4.63 vs 4.68 ± 6.55	–0.507 (–1.054 to 0.040)	*p* = 0.069
DELTA	5	31 vs 62	38.33 ± 20.36 vs 42.39 ± 15.50	–0.984 (–2.093 to 0.126)	*p* = 0.082
%DELTA	3	14 vs 41	9.91 ± 8.19 vs 10.25 ± 7.20	–0.167 (–0.972 to 0.638)	*p* = 0.685

Statistically significant meta-analysis *p*-values are shown in bold. TST, total sleep time; SOL, sleep onset latency; WASO, wake after sleep onset; REMT, rapid-eye-movement time; ST1, Stage 1 sleep time; ST2, Stage 2 sleep time; ST4, Stage 4 sleep time; %ST1, Stage 1 sleep percentage; %ST2, Stage 2 sleep percentage; %ST4, Stage 4 sleep percentage; DELTA, Delta sleep time; %DELTA, Delta sleep percentage; SE, sleep efficiency; REML, rapid-eye-movement latency; REMD, rapid-eye-movement density; RE, random effect.

**Table 7.** **Publication bias and funnel plot asymmetry (rank correlation and regression test) analysis**.

	Heterogeneity			Publication Bias			Funnel plot asymmetry	
SZA vs HC	Q	I^2^	*p*-value	Fail-Safe N (*p*-value)	Outlier	Overly influential	Rank correlation (*p*-value)	Regression test (*p*-value)
TST	2.797	0%	*p* = 0.424	48 (*p* < 0.001)	-	-	*p* = 0.7500	*p* = 0.4260
SOL	4.379	10.43%	*p* = 0.357	105 (*p* < 0.001)	-	Benson et al., 1980 [72]	*p* = 0.0833	*p* = 0.1477
WASO	2.262	6.62%	*p* = 0.323	19 (*p* < 0.001)	-	-	*p* = 1.0000	*p* = 0.8293
REMT	1.495	0%	*p* = 0.683	8 (*p* = 0.002)	-	-	*p* = 0.3333	*p* = 0.2678
%REM	1.722	0%	*p* = 0.787	0 (*p* = 0.399)	-	-	*p* = 0.4833	*p* = 0.3592
REML	1.369	0%	*p* = 0.849	9 (*p* = 0.003)	-	-	*p* = 0.8167	*p* = 0.8123
ST4	0.166	0%	*p* = 0.920	21 (*p* < 0.001)	-	-	*p* = 1.0000	*p* = 0.9619
%ST4	0.429	0%	*p* = 0.807	19 (*p* < 0.001)	-	-	*p* = 1.0000	*p* = 0.9100
DELTA	6.107	67.66%	*p* = 0.047	8 (*p* < 0.001)	Zarcone et al., 1995 [75]	-	*p* = 0.3333	*p* = 0.0874
	**Heterogeneity**			**Publication Bias**			**Funnel plot asymmetry**	
**SZA vs MDD**	**Q**	**I^2^**	***p*****-value**	**Fail-Safe N (*****p*****-value)**	**Outlier**	**Overly influential**	**Rank correlation (*****p*****-value)**	**Regression test (*****p*****-value)**
TST	21.237	80.04%	*p* = 0.002	0 (*p* = 0.431)	Kupfer & Foster, 1975 [77]	Kupfer & Foster, 1975 [77]	*p* = 0.7726	*p* = 0.1983
SOL	4.931	0%	*p* = 0.553	31 (*p* < 0.001)	-	-	*p* = 1.0000	*p* = 0.4002
SE	14.418	90.44%	*p* < 0.001	0 (*p* = 0.093)	Kupfer & Foster, 1975 [77]	-	*p* = 1.0000	*p* = 0.2274
WASO	19.199	88.48%	*p* < 0.001	0 (*p* = 0.137)	Kupfer & Foster, 1975 [77]	Kupfer & Foster, 1975 [77]	*p* = 1.0000	*p* = 0.0239*
REMT	20.393	80.67%	*p* = 0.002	0 (*p* = 0.349)	Kupfer & Foster, 1975 [77]	Kupfer & Foster, 1975 [77]	*p* = 0.2389	*p* = 0.0652
%REM	11.842	37.83%	*p* = 0.106	0 (*p* = 0.092)	Kupfer & Foster, 1975 [77]	-	*p* = 0.2751	*p* = 0.3701
REML	14.28	35.00%	*p* = 0.113	0 (*p* = 0.439)	-	-	*p* = 1.0000	*p* = 0.8657
REMD	0.152	0%	*p* = 0.927	0 (*p* = 0.342)	-	-	*p* = 1.0000	*p* = 0.7320
ST4	1.051	0%	*p* = 0.591	0 (*p* = 0.110)	-	-	*p* = 1.0000	*p* = 0.4381
%ST4	1.281	0%	*p* = 0.527	0 (*p* = 0.104)	-	-	*p* = 1.0000	*p* = 0.4131
DELTA	30.314	91.28%	*p* < 0.001	0 (*p* = 0.184)	Kupfer & Foster, 1975 [77]	-	*p* = 0.7194	*p* = 0.0379*
%DELTA	12.554	88.30%	*p* = 0.002	9 (*p* < 0.001)	Kupfer & Foster, 1975 [77]	-	*p* = 0.3333	*p* = 0.0005*
	**Heterogeneity**			**Publication Bias**			**Funnel plot asymmetry**	
**SZA vs SCZ**	**Q**	**I^2^**	***p*****-value**	**Fail-Safe N (*****p*****-value)**	**Outlier**	**Overly influential**	**Rank correlation (*****p*****-value)**	**Regression test (*****p*****-value)**
TST	15.949	66.73%	*p* = 0.014	0 (*p* = 0.378)	Reich et al., 1975 [76]	-	*p* = 1.0000	*p* = 0.6722
SOL	20.661	0%	*p* = 0.004	5 (*p* = 0.018)	Reich et al., 1975 [76]	-	*p* = 0.3988	*p* = 0.0003*
SE	14.999	93.61%	*p* < 0.001	7 (*p* = 0.002)	Reich et al., 1975 [76]	-	*p* = 1.0000	*p* = 0.0343*
WASO	14.377	77.93%	*p* = 0.006	10 (*p* = 0.003)	Reich et al., 1975 [76]	-	*p* = 0.4833	*p* = 0.8658
REMT	24.28	84.39%	*p* < 0.001	0 (*p* = 0.382)	Reich et al., 1975 [76]	Reich et al., 1975 [76]	*p* = 0.0690	*p* = 0.0005*
%REM	61.892	94.47%	*p* < 0.001	13 (*p* = 0.005)	Zarcone et al., 1987 [74]	Zarcone et al., 1987 [74]	*p* = 0.1789	*p* < 0.0001*
REML	41.256	93.60%	*p* < 0.001	7 (*p* = 0.014)	Reich et al., 1975 [76]	Reich et al., 1975 [76]	*p* = 0.3988	*p* < 0.0001*
REMD	21.365	89.27%	*p* < 0.001	23 (*p* < 0.001)	Benson et al., 1983 [80]	-	*p* = 1.0000	*p* = 0.0185*
ST1	3.517	44.12%	*p* = 0.172	1 (*p* = 0.043)	-	-	*p* = 0.3333	*p* = 0.0898
%ST1	0.161	0%	*p* = 0.923	0 (*p* = 0.289)	-	-	*p* = 0.3333	*p* = 0.7012
ST2	26.218	88.52%	*p* < 0.001	0 (*p* = 0.250)	Zarcone et al., 1995 [75]	-	*p* = 0.7500	*p* = 0.5116
%ST2	2.372	19.39%	*p* = 0.305	0 (*p* = 0.327)	-	-	*p* = 0.3333	*p* = 0.1563
ST4	0.862	0%	*p* = 0.650	1 (*p* = 0.049)	-	-	*p* = 1.0000	*p* = 0.9210
%ST4	1.101	0%	*p* = 0.577	1 (*p* = 0.034)	-	-	*p* = 1.0000	*p* = 0.7572
DELTA	16.348	80.35%	*p* = 0.003	16 (*p* < 0.001)	-	-	*p* = 0.0167*	*p* = 0.0001*
%DELTA	2.916	35.41%	*p* = 0.233	0 (*p* = 0.276)	-	-	*p* = 0.3333	*p* = 0.0878

*Asymmetry found.

### 3.2 Total Sleep Time

Drug-free SZA showed reduced TST (SMD = –1.43; *p* < 0.001) compared to HC. TST of drug-free SZA was not different from that of drug-free MDD (SMD = 0.0286; *p* = 0.948) and drug-free SCZ (SMD = –0.0519; *p* = 0.887).

### 3.3 Sleep Latency

Drug-free SZA showed prolonged SOL compared to both HC (SMD = 1.70; *p* < 0.001) and drug-free MDD (SMD = 0.684; *p* < 0.001). SOL of drug-free SZA did not differ from that of drug-free SCZ (SMD = 0.210; *p* = 0.266).

### 3.4 Sleep Efficiency

Drug-free SZA SE did not differ from that of drug-free MDD (SMD = 0.535; *p* = 0.556) and drug-free SCZ (SMD = –1.63; *p* = 0.301). It was not possible to compare drug-free SZA and HC SE due to lack of data.

### 3.5 Wakefulness Time During Sleep

Drug-free SZA showed increased WASO compared to HC (SMD = 1.20; *p* < 0.001). WASO of drug-free SZA did not differ from that of drug-free MDD (SMD = –0.385; *p* = 0.550) and drug-free SCZ (SMD = 0.664; *p* = 0.210).

### 3.6 REM Time

Reduced REMT was found in drug-free SZA compared to HC (SMD = –0.620; *p* = 0.006). REMT of drug-free SZA did not differ from that of drug-free MDD (SMD = 0.009; *p* = 0.984) and drug-free SCZ (SMD = 0.218; *p* = 0.688).

### 3.7 REM Percentage

Drug-free SZA %REM did not differ from that of HC (SMD = –0.0382; *p* = 0.843), drug-free MDD (SMD = –0.246; *p* = 0.277) and drug-free SCZ (SMD = 1.06; *p* = 0.244).

### 3.8 REM Latency

Drug-free SZA showed shortened REML compared to HC (SMD = –0.528; *p* = 0.007). REML of drug-free SZA did not differ from that of drug-free MDD (SMD = 0.0335; *p* = 0.870) and drug-free SCZ (SMD = –0.894; *p* = 0.263).

### 3.9 REM Density

Drug-free SZA REMD did not differ from that of drug-free MDD (SMD = –0.150; *p* = 0.582) and drug-free SCZ (SMD = 3.12; *p* = 0.081). It was not possible to compare drug-free SZA and HC REMD due to lack of data.

### 3.10 Stage 1 Sleep Time

Drug-free SZA ST1 did not differ from that of drug-free SCZ (SMD = 0.558; *p* = 0.216). It was not possible to compare drug-free SZA ST1 to that of HC and drug-free MDD due to lack of data.

### 3.11 Stage 1 Sleep Percentage

Drug-free SZA %ST1 did not differ from that of drug-free SCZ (SMD = 0.203; *p* = 0.516). It was not possible to compare drug-free SZA %ST1 to that of HC and drug-free MDD due to lack of data.

### 3.12 Stage 2 Sleep Time

Drug-free SZA ST2 did not differ from that of drug-free SCZ (SMD = –0.137; *p* = 0.874). It was not possible to compare drug-free SZA ST2 to that of HC and drug-free MDD due to lack of data.

### 3.13 Stage 2 Sleep Percentage

Drug-free SZA %ST2 did not differ from that of drug-free SCZ (SMD = –0.198; *p* = 0.583). It was not possible to compare drug-free SZA %ST2 to that of HC and drug-free MDD due to lack of data.

### 3.14 Stage 3 Sleep Time

It was not possible to perform a meta-analysis for ST3 in any case-control due to lack of data.

### 3.15 Stage 3 Sleep Percentage

It was not possible to perform a meta-analysis for %ST3 in any case-control due to lack of data.

### 3.16 Stage 4 Sleep Time

Drug-free SZA showed reduced ST4 compared to HC (SMD = –1.20; *p* < 0.001). ST4 of drug-free SZA did not differ from that of drug-free MDD (SMD = –0.348; *p* = 0.234) and drug-free SCZ (SMD = –0.461; *p* = 0.097).

### 3.17 Stage 4 Sleep Percentage

Drug-free SZA showed reduced %ST4 compared to HC (SMD = –1.14; *p* < 0.001). %ST4 of drug-free SZA did not differ from that of drug-free MDD (SMD = –0.357; *p* = 0.223) and drug-free SCZ (SMD = –0.507; *p* = 0.069).

### 3.18 Delta Sleep Time

Drug-free SZA DELTA did not differ from that of HC (SMD = –0.814; *p* = 0.080), drug-free MDD (SMD = 0.351; *p* = 0.609) and drug-free SCZ (SMD = –0.984; *p* = 0.082).

### 3.19 Delta Sleep Percentage

Drug-free SZA %DELTA did not differ from that of drug-free MDD (SMD = 1.16; *p* = 0.171) and drug-free SCZ (SMD = –0.167; *p* = 0.685). It was not possible to compare drug-free SZA %DELTA to that of HC due to lack of data.

## 4. Discussion

### 4.1 Previous Studies

Literature on sleep including samples with only SZA patients is limited, with most of the studies including SZA patients together with SCZ patients; therefore, it is difficult to clearly understand which sleep disturbances and abnormalities occur in SZA and their prevalence, as it is difficult to understand whether SZA sleep resembles more that of SCZ or that of affective disorders. The present work is the only systematic review and meta-analysis to date regarding sleep in SZA. Very few literature reviews on SZA have been performed, with each of them suggesting the necessity of more studies focused on these patients’ sleep [25,113]; however, case-control sleep studies providing clear, drug-free SZA patients sample can be found only in the 1975–1995 span.

In a previous meta-analysis on REM sleep in SCZ [111], the SZA case-control studies identified were excluded from the SCZ meta-analysis, and are now analyzed and discussed. The debate surrounding whether SZA exists as a separate nosography entity or not, as previously reported, might help understand the loss of interest towards this investigation.

Our meta-analysis results strengthen previous reports regarding sleep continuity fragmentation in SZA with decreased TST, increased SOL and WASO compared to HC. Sleep architecture analyses found decreased REMT and shortened REML, together with ST4 and %ST4 reduction. Nonetheless, in contrast with earlier reports, our meta-analysis did not find reduced DELTA (ST3+ST4) in SZA compared to HC.

We found significantly longer SOL in SZA compared to MDD, a result that was not consistently reported in previous studies. No differences were found between sleep parameters of SZA and SCZ.

According to our results and in line with more recent PSG studies [72,74,75], sleep variables do not allow to clearly characterize SZA patients from either SCZ or MDD patients. In the latest PSG study of SZA, although based on partially treated samples, Petit et al. [65] supported the idea that it might not be possible to differentiate SCZ, SZA, and BD patients by sleep parameters, as they found no PSG differences across these samples.

### 4.2 Clinical Implications

PSG parameters alone are not recommended for the differential diagnosis of SZA. The assessment of sleep disturbances as dimensional features (rather than categorical markers) is relevant for guiding adjunctive interventions targeting sleep quality.

Knowledge regarding the beneficial effect of drug treatment on the sleep of SZA patients is limited. Paliperidone, the only approved treatment for SZA in the United States, was reported to improve both subjective sleep quality [114] and sleep continuity [115].

### 4.3 Limitations and Future Perspectives

The majority of the meta-analyses performed in this study were based on datasets between three and ten, reflecting the limited availability of eligible studies. Meta-analyses, such as %DELTA in SCZ vs HC or those concerning the first two stages of sleep in SCZ vs MDD, could not be performed due to insufficient data. Most meta-analyses included fewer than five studies, below what has been suggested as a practical lower boundary for obtaining more stable random-effects meta-analytic estimates [116]. Post-hoc analysis revealed that the statistical power of most of the meta-analyses was low (<0.8), especially in those comparing SZA to MDD and to SCZ, substantially increasing the risk of type II error.

These constraints result in a partial characterization of sleep abnormalities in SZA, as several potentially relevant parameters could not be adequately examined. Therefore, the present meta-analysis should be considered exploratory. In addition, several analyses showed substantial heterogeneity, further limiting the strength of quantitative inferences and supporting more hypothesis-generating interpretation rather than definitive conclusions.

A major contributor to between-study heterogeneity is the marked demographic imbalance across samples. Most of the included studies recruited predominantly male participants, with female SZA patients being underrepresented, despite schizoaffective disorder being reported to occur more frequently in females. In addition, most samples were concentrated within a narrow age range (approximately 30–40 years), limiting generalizability across the lifespan and potentially increasing sensitivity to modest between-study age differences.

Diagnostic classification represents another important source of heterogeneity. In the meta-analyses, 8 of 9 studies used Research Diagnostic Criteria (RDC), while one study used DSM-II criteria. Although relatively consistent within the meta-analytic subset, the broader set of studies included in the systematic review shows greater variability in diagnostic frameworks across DSM and ICD versions. This inconsistency in diagnostic operationalization likely increases conceptual heterogeneity and reduces comparability across studies in the qualitative synthesis.

Although most studies applied a minimum washout period of ≥2 weeks, one study [75] used a shorter ≥2 days period. Residual pharmacological effects on sleep architecture cannot be entirely excluded, as several psychotropic agents may exert prolonged effects beyond such washout periods. However, given that only a single study deviated from the ≥2 week standard, its influence on the overall estimates is likely limited, although its contribution to residual heterogeneity cannot be formally quantified in the present dataset.

A further limitation concerns clinical heterogeneity within the MDD samples. Several included studies did not consistently distinguish between psychotic and non-psychotic forms of unipolar depression, preventing stratified analyses. Given that psychotic features may be associated with distinct sleep architecture alterations, the lack of diagnostic separation may have contributed to residual heterogeneity and limited the precision with which sleep findings can be attributed to specific depressive subtypes. Our meta-analysis found increased SOL in SZA compared with MDD; initial insomnia has been reported to be more severe in psychotic than non-psychotic depression [117], suggesting that prolonged SOL may be more closely related to psychotic symptomatology. This interpretation is further supported by the absence of SOL differences between SZA and SCZ in our analyses.

It must also be noted that the approach used to handle missing SDs represents a potential source of bias and may have influenced both effect size precision and between-study variability. Specifically, imputing missing SDs using weighted averages derived from studies within the same meta-analysis assumes homogeneity of variance across studies. Although this procedure allowed the inclusion of studies that otherwise would have been excluded due to incomplete reporting, it may have artificially constrained within-study variance in some cases while inflating it in others. This could have distorted the true magnitude of heterogeneity. For this reason, results involving imputed SDs should be interpreted with caution.

Our study did not permit a thorough investigation of DELTA in SZA compared to HC, as it was not possible to perform a meta-analysis with adequate statistical power of REMD in SZA compared to that in SCZ. This limitation is relevant given that REMD has consistently been reported as elevated in mood disorders [118,119,120,121], while early PSG study reported REMD levels in SZA and MDD exceeding those observed in HC and non-affective psychoses [71,72,73,74,75,76,77].

Given these limitations, the observed null findings should not be interpreted as evidence of equivalence between the studied populations, but rather as reflecting the limited and underpowered evidence base. It is nonetheless plausible that sleep macroarchitecture measures derived from standard PSG are not diagnosis-specific and therefore do not clearly differentiate SZA from other psychiatric conditions.

In this context, emerging research on sleep microarchitecture (e.g., sleep spindles) appears more sensitive to subtle group differences and may help clarify distinctions across conditions that can be conceptualized as lying along a psychosis-affective spectrum rather than representing discrete entities. Future research should also prioritize larger drug-free or minimally treated SZA cohorts to reduce the confounding effects of psychotropic medications on sleep architecture. Nonetheless, the effects of pharmacological treatment on sleep parameters, together with its clinical efficacy in SZA, should also be investigated. Longitudinal designs following patients across different illness phases and diagnostic transitions, especially given the high diagnostic shift in this population [122], may also help clarify the temporal stability and diagnostic specificity of PSG abnormalities. In addition, the relevance of REMD warrants further investigation, given its proposed utility as a clinical marker and its potential role in differentiating diagnostic subgroups [111,120,123,124].

Multimodal studies integrating PSG and electrophysiological measures with neuroimaging, as well as modern non-invasive digital sleep assessment tools such as actigraphy [125,126] and digital phenotyping [127,128] could improve understanding of the neurobiological mechanisms underlying sleep alterations across psychotic and affective disorders.

## 5. Conclusions

This systematic review and meta-analysis found that SZA is characterized by sleep disruption, including reduced TST, increased SOL and WASO, shortened REML, and reduced SWS compared to HC. However, these alterations do not allow to differentiate SZA from either SCZ or MDD, as most PSG parameters overlap across these conditions. Limited statistical power due to insufficient available data highlights the need for further studies with larger minimally treated samples to clarify the potential diagnostic specificity of sleep parameters in SZA.

## Data Availability

Data fully provided in **Supplementary Material**.

## Abbreviations

BD, Bipolar disorder; DELTA, Delta sleep time; %DELTA, Delta sleep percentage; EEG, Electroencephalography; HC, Healthy control; MDD, Major depressive disorder; NREM, Non-REM sleep; PRISMA, Preferred reporting items for systematic reviews and meta-analyses; PSG, Polysomnography; RDC, Research diagnostic criteria; REM, Rapid-eye-movement; REMD, Rapid-eye-movement density; REML, Rapid-eye-movement latency; REMT, Rapid-eye-movement time; %REM, Rapid-eye-movement percentage; SCZ, Schizophrenia; SD, Standard deviation; SE, Sleep efficiency; SOL, Sleep onset latency; ST1, Stage 1 sleep time; ST2, Stage 2 sleep time; ST4, Stage 4 sleep time; %ST1, Stage 1 sleep percentage; %ST2, Stage 2 sleep percentage; %ST4, Stage 4 sleep percentage; SWS, Slow-wave sleep; SZA, Schizoaffective disorder; TST, Total sleep time; WASO, Wake after sleep onset; WoS, Web of Science.

## References

[1] Jäger M, Haack S, Becker T, Frasch K (2011). Schizoaffective disorder--an ongoing challenge for psychiatric nosology. European Psychiatry.

[2] Malaspina D, Owen MJ, Heckers S, Tandon R, Bustillo J, Schultz S (2013). Schizoaffective Disorder in the DSM-5. Schizophrenia Research.

[3] Peterson DL, Webb CA, Keeley JW, Gaebel W, Zielasek J, Rebello TJ (2019). The reliability and clinical utility of ICD-11 schizoaffective disorder: A field trial. Schizophrenia Research.

[4] Sovner RD, McHugh PR (1976). Bipolar course in schizo-affective illness. Biological Psychiatry.

[5] Rosenthal NE, Rosenthal LN, Stallone F, Dunner DL, Fieve RR (1980). Toward the validation of RDC schizoaffective disorder. Archives of General Psychiatry.

[6] Cheniaux E, Landeira-Fernandez J, Lessa Telles L, Lessa JLM, Dias A, Duncan T (2008). Does schizoaffective disorder really exist? A systematic review of the studies that compared schizoaffective disorder with schizophrenia or mood disorders. Journal of Affective Disorders.

[7] Rink L, Pagel T, Franklin J, Baethge C (2016). Characteristics and heterogeneity of schizoaffective disorder compared with unipolar depression and schizophrenia - a systematic literature review and meta-analysis. Journal of Affective Disorders.

[8] Miller JN, Black DW (2019). Schizoaffective disorder: A review. Annals of Clinical Psychiatry.

[9] Abrams DJ, Rojas DC, Arciniegas DB (2008). Is schizoaffective disorder a distinct categorical diagnosis? A critical review of the literature. Neuropsychiatric Disease and Treatment.

[10] Angst J, Felder W, Lohmeyer B (1980). Course of schizoaffective psychoses: results of a followup study. Schizophrenia Bulletin.

[11] Marneros A, Deister A, Rohde A (1990). Psychopathological and social status of patients with affective, schizophrenic and schizoaffective disorders after long-term course. Acta Psychiatrica Scandinavica.

[12] Scully PJ, Owens JM, Kinsella A, Waddington JL (2004). Schizophrenia, schizoaffective and bipolar disorder within an epidemiologically complete, homogeneous population in rural Ireland: small area variation in rate. Schizophrenia Research.

[13] Malhi GS, Green M, Fagiolini A, Peselow ED, Kumari V (2008). Schizoaffective disorder: diagnostic issues and future recommendations. Bipolar Disorders.

[14] Marneros A, Röttig S, Wenzel A, Blöink R, Brieger P (2004). Affective and schizoaffective mixed states. European Archives of Psychiatry and Clinical Neuroscience.

[15] Hor K, Taylor M (2010). Suicide and schizophrenia: a systematic review of rates and risk factors. Journal of Psychopharmacology (Oxford, England).

[16] Harrow M, Grossman LS, Herbener ES, Davies EW (2000). Ten-year outcome: patients with schizoaffective disorders, schizophrenia, affective disorders and mood-incongruent psychotic symptoms. The British Journal of Psychiatry.

[17] Pinna F, Sanna L, Perra V, Pisu Randaccio R, Diana E, Carpiniello B (2014). Long-term outcome of schizoaffective disorder. Are there any differences with respect to schizophrenia?. Rivista Di Psichiatria.

[18] Kraepelin E (1899). Psychiatrie: Ein Lehrbuch für Studierende und Ärzte. 6th edn.

[19] Angst J (2002). Historical aspects of the dichotomy between manic-depressive disorders and schizophrenia. Schizophrenia Research.

[20] Kasanin J (1993). The acute schizoaffective psychoses. 1933. The American Journal of Psychiatry.

[21] Santelmann H, Franklin J, Bußhoff J, Baethge C (2015). Test-retest reliability of schizoaffective disorder compared with schizophrenia, bipolar disorder, and unipolar depression--a systematic review and meta-analysis. Bipolar Disorders.

[22] Wilson JE, Nian H, Heckers S (2014). The schizoaffective disorder diagnosis: a conundrum in the clinical setting. European Archives of Psychiatry and Clinical Neuroscience.

[23] Paul T, Javed S, Karam A, Loh H, Ferrer GF (2021). A Misdiagnosed Case of Schizoaffective Disorder With Bipolar Manifestations. Cureus.

[24] Wy TJP, Saadabadi A (2025). Schizoaffective Disorder. StatPearls.

[25] Meltzer HY (1984). Schizoaffective disorder: is the news of its nonexistence premature? Editor's introduction. Schizophrenia Bulletin.

[26] Tsuang MT, Simpson JC (1984). Schizoaffective disorder: concept and reality. Schizophrenia Bulletin.

[27] Lapierre YD (1994). Schizophrenia and manic-depression: separate illnesses or a continuum?. Canadian Journal of Psychiatry. Revue Canadienne De Psychiatrie.

[28] Jäger M, Bottlender R, Strauss A, Möller HJ (2004). Fifteen-year follow-up of ICD-10 schizoaffective disorders compared with schizophrenia and affective disorders. Acta Psychiatrica Scandinavica.

[29] Lake CR, Hurwitz N (2007). Schizoaffective disorder merges schizophrenia and bipolar disorders as one disease--there is no schizoaffective disorder. Current Opinion in Psychiatry.

[30] Cheniaux E, Landeira-Fernandez J, Versiani M (2009). The diagnoses of schizophrenia, schizoaffective disorder, bipolar disorder and unipolar depression: interrater reliability and congruence between DSM-IV and ICD-10. Psychopathology.

[31] Kantrowitz JT, Citrome L (2011). Schizoaffective disorder: a review of current research themes and pharmacological management. CNS Drugs.

[32] Pavlichenko A, Petrova N, Stolyarov A (2024). The Modern Concept of Schizoaffective Disorder: A Narrative Review. Consortium Psychiatricum.

[33] Madre M, Canales-Rodríguez EJ, Ortiz-Gil J, Murru A, Torrent C, Bramon E (2016). Neuropsychological and neuroimaging underpinnings of schizoaffective disorder: a systematic review. Acta Psychiatrica Scandinavica.

[34] Gotra MY, Hill SK, Gershon ES, Tamminga CA, Ivleva EI, Pearlson GD (2020). Distinguishing patterns of impairment on inhibitory control and general cognitive ability among bipolar with and without psychosis, schizophrenia, and schizoaffective disorder. Schizophrenia Research.

[35] Dehelean L, Romosan AM, Bucatos BO, Papava I, Balint R, Bortun AMC (2021). Social and Neurocognitive Deficits in Remitted Patients with Schizophrenia, Schizoaffective and Bipolar Disorder. Healthcare (Basel, Switzerland).

[36] Radonić E, Rados M, Kalember P, Bajs-Janović M, Folnegović-Smalc V, Henigsberg N (2011). Comparison of hippocampal volumes in schizophrenia, schizoaffective and bipolar disorder. Collegium Antropologicum.

[37] Ivleva EI, Bidesi AS, Thomas BP, Meda SA, Francis A, Moates AF (2012). Brain gray matter phenotypes across the psychosis dimension. Psychiatry Research.

[38] Ivleva EI, Bidesi AS, Keshavan MS, Pearlson GD, Meda SA, Dodig D (2013). Gray matter volume as an intermediate phenotype for psychosis: Bipolar-Schizophrenia Network on Intermediate Phenotypes (B-SNIP). The American Journal of Psychiatry.

[39] Amann BL, Canales-Rodríguez EJ, Madre M, Radua J, Monte G, Alonso-Lana S (2016). Brain structural changes in schizoaffective disorder compared to schizophrenia and bipolar disorder. Acta Psychiatrica Scandinavica.

[40] Lynham AJ, Cleaver SL, Jones IR, Walters JTR (2022). A meta-analysis comparing cognitive function across the mood/psychosis diagnostic spectrum. Psychological Medicine.

[41] Rieder RO, Mann LS, Weinberger DR, van Kammen DP, Post RM (1983). Computed tomographic scans in patients with schizophrenia, schizoaffective, and bipolar affective disorder. Archives of General Psychiatry.

[42] Hager B, Yang AC, Brady R, Meda S, Clementz B, Pearlson GD (2017). Neural complexity as a potential translational biomarker for psychosis. Journal of Affective Disorders.

[43] Du Y, Hao H, Wang S, Pearlson GD, Calhoun VD (2020). Identifying commonality and specificity across psychosis sub-groups via classification based on features from dynamic connectivity analysis. NeuroImage. Clinical.

[44] Mazza MG, Capellazzi M, Tagliabue I, Lucchi S, Rossetti A, Clerici M (2019). Neutrophil-lymphocyte, monocyte-lymphocyte and platelet-lymphocyte ratio in schizoaffective disorder compared to schizophrenia. General Hospital Psychiatry.

[45] Jeannin J, Romeo B, Lestra V, Martelli C, Amirouche A, Benyamina A (2026). Could peripheral immunological markers discriminate schizophrenia and schizoaffective disorders from bipolar disorder?. Journal of Psychiatric Research.

[46] Cardno AG, Marshall EJ, Coid B, Macdonald AM, Ribchester TR, Davies NJ (1999). Heritability estimates for psychotic disorders: the Maudsley twin psychosis series. Archives of General Psychiatry.

[47] Coryell W, Zimmerman M (1988). The heritability of schizophrenia and schizoaffective disorder. A family study. Archives of General Psychiatry.

[48] Lapensée MA (1992). A review of schizoaffective disorder: I. Current concepts. Canadian Journal of Psychiatry. Revue Canadienne De Psychiatrie.

[49] Laursen TM, Labouriau R, Licht RW, Bertelsen A, Munk-Olsen T, Mortensen PB (2005). Family history of psychiatric illness as a risk factor for schizoaffective disorder: a Danish register-based cohort study. Archives of General Psychiatry.

[50] Fallin MD, Lasseter VK, Avramopoulos D, Nicodemus KK, Wolyniec PS, McGrath JA (2005). Bipolar I disorder and schizophrenia: a 440-single-nucleotide polymorphism screen of 64 candidate genes among Ashkenazi Jewish case-parent trios. American Journal of Human Genetics.

[51] Kendler KS, Ohlsson H, Sundquist J, Sundquist K (2025). Profiles of Genetic Risks for Psychotic Disorders. JAMA Psychiatry.

[52] Marneros A (2010). The psychotic "continuum". Psychiatrike = Psychiatriki.

[53] Guloksuz S, van Os J (2018). The slow death of the concept of schizophrenia and the painful birth of the psychosis spectrum. Psychological Medicine.

[54] Gama Marques J, Ouakinin S (2021). Schizophrenia-schizoaffective-bipolar spectra: an epistemological perspective. CNS Spectrums.

[55] Muñoz-Negro JE, Cuadrado L, Cervilla JA (2019). Current Evidences on Psychopharmacology of Schizoaffective Disorder. Actas Espanolas De Psiquiatria.

[56] Canuso CM, Lindenmayer JP, Kosik-Gonzalez C, Turkoz I, Carothers J, Bossie CA (2010). A randomized, double-blind, placebo-controlled study of 2 dose ranges of paliperidone extended-release in the treatment of subjects with schizoaffective disorder. The Journal of Clinical Psychiatry.

[57] Canuso CM, Schooler N, Carothers J, Turkoz I, Kosik-Gonzalez C, Bossie CA (2010). Paliperidone extended-release in schizoaffective disorder: a randomized, controlled study comparing a flexible dose with placebo in patients treated with and without antidepressants and/or mood stabilizers. Journal of Clinical Psychopharmacology.

[58] Palmese LB, DeGeorge PC, Ratliff JC, Srihari VH, Wexler BE, Krystal AD (2011). Insomnia is frequent in schizophrenia and associated with night eating and obesity. Schizophrenia Research.

[59] Kaskie RE, Graziano B, Ferrarelli F (2017). Schizophrenia and sleep disorders: links, risks, and management challenges. Nature and Science of Sleep.

[60] Laskemoen JF, Simonsen C, Büchmann C, Barrett EA, Bjella T, Lagerberg TV (2019). Sleep disturbances in schizophrenia spectrum and bipolar disorders - a transdiagnostic perspective. Comprehensive Psychiatry.

[61] Alam A, Chengappa KNR, Ghinassi F (2012). Screening for obstructive sleep apnea among individuals with severe mental illness at a primary care clinic. General Hospital Psychiatry.

[62] Annamalai A, Palmese LB, Chwastiak LA, Srihari VH, Tek C (2015). High rates of obstructive sleep apnea symptoms among patients with schizophrenia. Psychosomatics.

[63] Poulin J, Chouinard S, Pampoulova T, Lecomte Y, Stip E, Godbout R (2010). Sleep habits in middle-aged, non-hospitalized men and women with schizophrenia: a comparison with healthy controls. Psychiatry Research.

[64] Miller BJ, Parker CB, Rapaport MH, Buckley PF, McCall WV (2019). Insomnia and suicidal ideation in nonaffective psychosis. Sleep.

[65] Petit JM, Strippoli MPF, Stephan A, Ranjbar S, Haba-Rubio J, Solelhac G (2022). Sleep spindles in people with schizophrenia, schizoaffective disorders or bipolar disorders: a pilot study in a general population-based cohort. BMC Psychiatry.

[66] Tanrıseven E, Sağtaş E, Topak OZ, Gündüz M, Dogruyol CA (2026). An evaluation of the differences in thalamic nuclei and subnuclei among patients with schizophrenia and schizoaffective disorder. Psychiatry Research. Neuroimaging.

[67] Castelnovo A, D'Agostino A, Mayeli A, Albantakis L, Tononi G, Ferrarelli F (2025). Sleep Spindle Abnormalities as Neurophysiological Biomarkers of Schizophrenia Spectrum Disorders: From Cellular Mechanisms and Neural Circuits to Clinical Implications. Biological Psychiatry.

[68] Ferrarelli F, Tononi G (2017). Reduced sleep spindle activity point to a TRN-MD thalamus-PFC circuit dysfunction in schizophrenia. Schizophrenia Research.

[69] Dimitriades ME, Schumacher E, Arudchelvam J, Fattinger S, Kurth S, Pugin F (2025). The infraslow fluctuation of sigma power during sleep in young individuals with schizophrenia. Schizophrenia Research.

[70] Hauri P, Hawkins DR (1973). Alpha-delta sleep. Electroencephalography and Clinical Neurophysiology.

[71] Lahmeyer HW, Val E, Gaviria FM, Prasad RB, Pandey GN, Rodgers P (1988). EEG sleep, lithium transport, dexamethasone suppression, and monoamine oxidase activity in borderline personality disorder. Psychiatry Research.

[72] Benson KL, Berger PA, Zarcone VP (1980). Sleep stage measures of psychopathology using Research Diagnostic Criteria. Sleep Research.

[73] Benson KL, Zarcone VP (1985). Testing the REM sleep phasic event intrusion hypothesis of schizophrenia. Psychiatry Research.

[74] Zarcone VP, Benson KL, Berger PA (1987). Abnormal rapid eye movement latencies in schizophrenia. Archives of General Psychiatry.

[75] Zarcone VP, Benson KL (1995). Middle ear muscle activity (MEMA) in schizophrenia using a noninvasive technique. Sleep.

[76] Reich L, Weiss BL, Coble P, McPartland R, Kupfer DJ (1975). Sleep disturbance in schizophrenia. A revisit. Archives of General Psychiatry.

[77] Kupfer DJ, Foster FG (1975). The sleep of psychotic patients: does it all look alike?. Research Publications - Association for Research in Nervous and Mental Disease.

[78] Kupfer DJ, Broudy D, Spiker DG, Neil JF, Coble PA (1979). EEG sleep and affective psychoses: I. Schizoaffective disorders. Psychiatry Research.

[79] Coble PA, Kupfer DJ, Shaw DH (1981). Distribution of REM latency in depression. Biological Psychiatry.

[80] Benson KL, Zarcone VP, Faull KF, Barchas JD, Berger PA (1983). REM sleep eye movement activity and CSF concentrations of 5-hydroxyindoleacetic acid in psychiatric patients. Psychiatry Research.

[81] Kupfer DJ, Wyatt RJ, Synder F, Davis JM (1971). Chlorpromazine and sleep in psychiatric patients. Archives of General Psychiatry.

[82] Gillin JC, van Kammen DP, Post R, Bunney WE (1977). Effects of prolonged administration of pimozide on sleep-EEG patterns in psychiatric patients. Communications in Psychopharmacology.

[83] Gillin JC, van Kammen DP, Bunney WE (1978). Pimozide attentuates d-amphetamine-induced sleep changes in man. Life Sciences.

[84] Keshavan MS, Reynolds CF, Miewald JM, Montrose DM (1996). A longitudinal study of EEG sleep in schizophrenia. Psychiatry Research.

[85] Benson KL, Sullivan EV, Lim KO, Lauriello J, Zarcone VP, Pfefferbaum A (1996). Slow wave sleep and computed tomographic measures of brain morphology in schizophrenia. Psychiatry Research.

[86] Nishino S, Mignot E, Benson KL, Zarcone VP (1998). Cerebrospinal fluid prostaglandins and corticotropin releasing factor in schizophrenics and controls: relationship to sleep architecture. Psychiatry Research.

[87] Keshavan MS, Reynolds CF, Miewald MJ, Montrose DM, Sweeney JA, Vasko RC (1998). Delta sleep deficits in schizophrenia: evidence from automated analyses of sleep data. Archives of General Psychiatry.

[88] Rotenberg P, Indurski R, Kimhi J, Hadjez Y, Gutman E, Shamir Y (2000). The relationship between objective sleep variables and subjective sleep estimation in schizophrenia. International Journal of Psychiatry in Clinical Practice.

[89] Armitage R, Cole D, Suppes T, Ozcan ME (2004). Effects of clozapine on sleep in bipolar and schizoaffective disorders. Progress in Neuro-psychopharmacology & Biological Psychiatry.

[90] Kluge M, Schacht A, Himmerich H, Rummel-Kluge C, Wehmeier PM, Dalal M (2014). Olanzapine and clozapine differently affect sleep in patients with schizophrenia: results from a double-blind, polysomnographic study and review of the literature. Schizophrenia Research.

[91] Göder R, Graf A, Ballhausen F, Weinhold S, Baier PC, Junghanns K (2015). Impairment of sleep-related memory consolidation in schizophrenia: relevance of sleep spindles?. Sleep Medicine.

[92] Kaskie RE, Graziano B, Ferrarelli F (2019). Topographic deficits in sleep spindle density and duration point to frontal thalamo-cortical dysfunctions in first-episode psychosis. Journal of Psychiatric Research.

[93] Gerstenberg M, Furrer M, Tesler N, Franscini M, Walitza S, Huber R (2020). Reduced sleep spindle density in adolescent patients with early-onset schizophrenia compared to major depressive disorder and healthy controls. Schizophrenia Research.

[94] Denis D, Baran B, Mylonas D, Spitzer C, Raymond N, Talbot C (2024). Sleep oscillations and their relations with sleep-dependent memory consolidation in early course psychosis and first-degree relatives. Schizophrenia Research.

[95] Gillin JC, Schulz SC, van Kammen DP, Balow JE, Bunney WE (1981). EEG sleep patterns in schizophrenic patients undergoing hemodialysis. Psychiatry Research.

[96] Glassman JN, Darko D, Gillin JC (1986). Medication-induced somnambulism in a patient with schizoaffective disorder. The Journal of Clinical Psychiatry.

[97] Keshavan MS, Reynolds CF, Ganguli R, Brar J, Houck P (1991). Electroencephalographic sleep and cerebral morphology in functional psychoses: a preliminary study with computed tomography. Psychiatry Research.

[98] Keshavan MS, Reynolds CF, Montrose D, Miewald J, Kupfer DJ (1992). Trait-like abnormalities in the sleep of patients with psychoses. Clinical Neuropharmacology.

[99] Douglass AB, Shipley JE, Haines RF, Scholten RC, Dudley E, Tapp A (1993). Schizophrenia, narcolepsy, and HLA-DR15, DQ6. Biological Psychiatry.

[100] Keshavan MS, Reynolds CF, Montrose D, Miewald J, Downs C, Sabo EM (1994). Sleep and suicidality in psychotic patients. Acta Psychiatrica Scandinavica.

[101] Keshaven MS, Reynolds CF, Miewald J, Montrose D (1995). Slow-wave sleep deficits and outcome in schizophrenia and schizoaffective disorder. Acta Psychiatrica Scandinavica.

[102] Baandrup L, Glenthøj BY, Jennum PJ (2016). Objective and subjective sleep quality: Melatonin versus placebo add-on treatment in patients with schizophrenia or bipolar disorder withdrawing from long-term benzodiazepine use. Psychiatry Research.

[103] Yamashita H, Horiguchi J, Mizuno S, Kuramoto Y, Yamawaki S, Inami Y (1999). A case of neuroleptic-induced unilateral akathisia with periodic limb movements in the opposite side during sleep. Psychiatry and Clinical Neurosciences.

[104] Wetter TC, Brunner J, Bronisch T (2002). Restless legs syndrome probably induced by risperidone treatment. Pharmacopsychiatry.

[105] Weinhold SL, Lechinger J, Timm N, Hansen A, Ngo HVV, Göder R (2022). Auditory stimulation in-phase with slow oscillations to enhance overnight memory consolidation in patients with schizophrenia?. Journal of Sleep Research.

[106] Giedke H, Geilenkirchen R, Hauser M (1992). The timing of partial sleep deprivation in depression. Journal of Affective Disorders.

[107] Keshavan MS, Mahadik SP, Reynolds CF, Mukerjee S, Korenovsky A, Houck P (1992). Plasma cholinesterase isozymes and REM latency in schizophrenia. Psychiatry Research.

[108] Itil TM, Hsu W, Holden JM, Gannon P (1970). Digital computer sleep prints in lobotomized and nonlobotomized schizophrenics. Biological psychiatry.

[109] Antrobus JS, Dement W, Fisher C (1964). Patterns of dreaming recall: An EEG study. The Journal of Abnormal and Social Psychology.

[110] ITIL TM (1965). PENTOTHAL INDUCED CHANGES IN EEG AS A PROGNOSTIC INDEX IN DRUGS THERAPY OF PSYCHOTIC PATIENTS. The American Journal of Psychiatry.

[111] Morra D, Barbato G (2025). REM sleep in schizophrenia: a systematic review and meta-analysis. Sleep Medicine Reviews.

[112] Higgins JPT, Thomas J, Chandler J, Cumpston M, Li T, Page MJ, Higgins JPT, Thomas J, Chandler J, Cumpston M, Li T, Page MJ (2024). Cochrane handbook for systematic reviews of interventions.

[113] Clayton PJ (1982). Schizoaffective disorders. The Journal of Nervous and Mental Disease.

[114] Kongsakon R, Thavichachart N, Chung KF, Lim L, Azucena B, Rondain E (2017). Evaluation of sleep profile in schizophrenia patients treated with extended-release paliperidone: an open-label prospective study in Southeast Asia. Psychology Research and Behavior Management.

[115] Luthringer R, Staner L, Noel N, Muzet M, Gassmann-Mayer C, Talluri K (2007). A double-blind, placebo-controlled, randomized study evaluating the effect of paliperidone extended-release tablets on sleep architecture in patients with schizophrenia. International Clinical Psychopharmacology.

[116] Jackson D, Turner R (2017). Power analysis for random-effects meta-analysis. Research Synthesis Methods.

[117] Thase ME, Kupfer DJ, Ulrich RF (1986). Electroencephalographic sleep in psychotic depression. A valid subtype?. Archives of General Psychiatry.

[118] Kupfer DJ, Heninger GR (1972). REM activity as a correlate of mood changes throughout the night. Electroencephalographic sleep patterns in a patient with a 48-hour cyclic mood disorder. Archives of General Psychiatry.

[119] Sitaram N, Nurnberger JI, Gershon ES, Gillin JC (1982). Cholinergic regulation of mood and REM sleep: potential model and marker of vulnerability to affective disorder. The American Journal of Psychiatry.

[120] Kheirkhah M, Duncan WC, Yuan Q, Wang PR, Jamalabadi H, Leistritz L (2025). REM density predicts rapid antidepressant response to ketamine in individuals with treatment-resistant depression. Neuropsychopharmacology.

[121] Del Giudice R, Stefanelli R, Donati F, Nisticò V, Casetta C, Castelnovo A (2026). Elevated gamma activity in left frontotemporal regions preceding sleep signals emotional arousal in Bipolar Disorders: Insights from a high-density EEG investigation. Journal of Affective Disorders.

[122] Santelmann H, Franklin J, Bußhoff J, Baethge C (2016). Diagnostic shift in patients diagnosed with schizoaffective disorder: a systematic review and meta-analysis of rediagnosis studies. Bipolar Disorders.

[123] Saleh C, Diederich NJ, Schroeder LA, Schmidt M, Rüegg S, Khatami R (2022). Time to reconsider REM density in sleep research. Clinical Neurophysiology.

[124] Barbato G (2023). Is REM Density a Measure of Arousal during Sleep?. Brain Sciences.

[125] Pieters LE, Deenik J, Hoogendoorn AW, van Someren EJW, van Harten PN (2025). Sleep and physical activity patterns in relation to daily-life symptoms in psychosis: An actigraphy and experience sampling study. Psychiatry Research.

[126] Thelagathoti RK, Siu KC, Ali HH, Fernando RM (2026). Integrated Analysis of Circadian and Sleep Signatures in Depression and Schizophrenia Using Multi-Day Actigraphy. Bioengineering (Basel, Switzerland).

[127] Garyfalli V, Kalisperakis E, Smyrnis A, Lazaridi M, Karantinos T, Mantas A (2025). Smartwatch-Derived Digital Phenotypes Relate to Psychopathology Dimensions in Patients With Psychotic Spectrum Disorders: Longitudinal Observational Study. JMIR Mental Health.

[128] Vecchio I, Mifsud L, Castro E Almeida S, Passecker J (2025). Diagnostic digital phenotyping in schizophrenia-spectrum disorders: a systematic review. NPJ Digital Medicine.

