# Supplementary material for: Polysomnographic Parameters in Schizoaffective Disorder: A Systematic Review and Meta-Analysis
Source: Alpha Psychiatry. 2026 Aug 19;27(4):52900. doi: 10.31083/AP52900 (PMC13540051; doi:10.31083/AP52900)
Supplement: Supplementary file 1 [file 2757-8038-27-4-52900-s1.zip › Supplementary Material/Supplementary Material.pdf]

Study	N. (SZA)/N. (HC)	Age (SZA/HC)	Males % (SZA/HC)	Diagnosis (SZA/HC)	Treatment
**Benson et al., 1980**	9/19	?/?	?/?	Schizoaffective disorder/Healthy	Drug-free (≥2 weeks)
**Benson et al., 1985**	8/13	33/29.7	100/100	Schizoaffective disorder/Healthy	Drug-free (≥2 weeks)
**Benson et al., 1985**	9/19	33.4/30.2	100/100	Schizoaffective disorder/Healthy	Drug-free (≥2 weeks)
**Zarcone et al., 1987**	8/18	33.8/30.3	100/100	Schizoaffective disorder/Healthy	Drug-free (≥2 weeks)
**Zarcone et al., 1995**	8/10	34.5/32.3	100/100	Schizoaffective disorder/Healthy	Drug-free (2 days)

Study	N. SZA	N. HC	REMT (SZA)	SD	REMT (HC)	SD	%REM (SZA)	SD	%REM (HC)	SD	REML (SZA)	SD	REML (HC)	SD	REMD (SZA)	SD	REMD (HC)	SD
**Benson et al., 1980**	9	19					23.66		22.38		75.98		96.66					
**Benson et al., 1985**	8	13	68.65	27.48	95.30	28.20	18.35	7.65	22.40	7.45	74.20	41.45	89.90	40.50				
**Benson et al., 1985**	9	19	92.30	38.80	98.90	21.00	22.70	7.85	22.70	5.40	73.80	24.65	82.20	32.90				
**Zarcone et al., 1987**	8	18	91.16	20.19	101.62	13.17	22.98	4.20	22.57	3.16	53.46	14.90	88.60	42.79				
**Zarcone et al., 1995**	8	10	65.10		86.40		20.20	4.00	20.80	4.50	52.20	32.80	65.30	16.40				

Study	N. (SZA)/N. (MDD)	Age (SZA/MDD)	Males % (SZA/MDD)	Diagnosis (SZA/MDD)	Treatment
**Kupfer & Foster, 1975**	6/9	38.8/58.2	50/?	Schizoaffective disorder/Psychotic depression	Drug-free (≥2 weeks)
**Kupfer & Foster, 1975**	6/25	38.8/41.1	50/?	Schizoaffective disorder/MDD	Drug-free (≥2 weeks)
**Kupfer et al., 1979**	12/29	44.4/48.1	42/31	Schizoaffective disorder/Psychotic depression	Drug-free (≥2 weeks)
**Benson et al., 1980**	9/6	?/?	?/?	Schizoaffective disorder/MDD	Drug-free (≥2 weeks)
**Coble et al., 1981**	5/17	34.8/40.1	60/35	Schizoaffective disorder/<br>Psychotic and nonpsychotic depression	Drug-free (2 weeks)
**Benson et al., 1983**	2/5	?/?	100/100	Schizoaffective disorder/MDD	Drug-free (≥2 weeks)
**Benson et al., 1985**	8/10	33/40.3	100/100	Schizoaffective disorder/MDD	Drug-free (≥2 weeks)
**Benson et al., 1985**	9/7	33.4/37.7	100/100	Schizoaffective disorder/MDD	Drug-free (≥2 weeks)
**Zarcone et al., 1987**	8/12	33.8/41.33	100/100	Schizoaffective disorder/MDD	Drug-free (≥2 weeks)
**Zarcone et al., 1995**	8/11	34.5/37.2	100/100	Schizoaffective disorder/MDD	Drug-free (2 days)

Study	N. SZA	N. MDD	REMT (SZA)	SD	REMT (MDD)	SD	%REM (SZA)	SD	%REM (MDD)	SD	REML (SZA)	SD	REML (MDD)	SD	REMD (SZA)	SD	REMD (MDD)	SD
**Kupfer & Foster, 1975**	6	9	81.00	10.50	51.30	7.00	23.20	1.90	19.60	2.40	31.70	5.70	36.00	7.20	2.33		2.35	
**Kupfer & Foster, 1975**	6	25									31.70	5.70	40.91	6.05				
**Kupfer et al., 1979**	12	29	47.22		57.24		17.70	6.60	21.10	5.30	23.41	27.90	41.20	33.40	1.34	0.71	1.55	0.95
**Benson et al., 1980**	9	6					23.66		30.23		75.98		49.33					
**Coble et al., 1981**	5	17									49.87		46.04					
**Benson et al., 1983**	2	5	90.20		106.66		22.96		24.97		68.25		62.28		2.62		2.61	
**Benson et al., 1985**	8	10	68.65	27.48	89.40	53.70	18.35	7.65	19.90	11.95	74.20	41.45	63.60	17.80				
**Benson et al., 1985**	9	7	92.30	38.80	109.60	52.70	22.70	7.85	26.30	14.30	73.80	24.65	44.30	65.70				
**Zarcone et al., 1987**	8	12	91.16	20.19	105.12	32.78	22.98	4.20	25.28	7.10	53.46	14.90	48.77	24.46				
**Zarcone et al., 1995**	8	11	65.10		83.25		20.20	4.00	21.80	3.90	52.20	32.80	45.50	22.10				

Study	N. (SZA)/N. (SCZ)	Age (SZA/SCZ)	Males % (SZA/SCZ)	Diagnosis (SZA/SCZ)	Treatment
**Reich et al., 1975**	3/14	38.8/20.8	50/57	Schizoaffective disorder/Schizophrenia (acute)	Drug-free (≥2 weeks)
**Reich et al., 1975**	3/9	38.8/19	50/11	Schizoaffective disorder/Schizophrenia (latent)	Drug-free (≥2 weeks)
**Benson et al., 1980**	9/9	?/?	?/?	Schizoaffective disorder/Schizophrenia	Drug-free (≥2 weeks)
**Benson et al., 1983**	2/7	?/?	100/100	Schizoaffective disorder/Schizophrenia	Drug-free (≥2 weeks)
**Benson et al., 1985**	8/11	33/27	100/100	Schizoaffective disorder/Schizophrenia	Drug-free (≥2 weeks)
**Benson et al., 1985**	9/8	33.4/26.6	100/100	Schizoaffective disorder/Schizophrenia	Drug-free (≥2 weeks)
**Zarcone et al., 1987**	8/12	33.8/26.2	100/100	Schizoaffective disorder/Schizophrenia	Drug-free (≥2 weeks)
**Zarcone et al., 1995**	8/18	34.5/33.1	100/100	Schizoaffective disorder/Schizophrenia	Drug-free (2 days)

Study	N. SZA	N. SCZ	REMT (SZA)	SD	REMT (SCZ)	SD	%REM (SZA)	SD	%REM (SCZ)	SD	REML (SZA)	SD	REML (SCZ)	SD	REMD (SZA)	SD	REMD (SCZ)	SD
**Reich et al., 1975**	3	14	81	10.5	54	5.6	23.20	1.90	17.40	1.60	31.70	5.70	97.90	11.00	2.20	0.20	1.60	0.10
**Reich et al., 1975**	3	9	81	10.5	74.8	5.6	23.20	1.90	20.00	1.40	31.70	5.70	81.00	12.00	2.20	0.20	1.50	0.10
**Benson et al., 1980**	9	9					23.66		25.44		75.98		66.20					
**Benson et al., 1983**	2	7	90.20		100.37		22.96		26.01		68.25		50.89		2.62		2.66	
**Benson et al., 1985**	8	11	68.65	27.48	98.00	40.30	18.35	7.65	25.85	5.70	74.20	41.45	62.90	41.00				
**Benson et al., 1985**	9	8	92.30	38.80	101.70	19.45	22.70	7.85	24.45	4.78	73.80	24.65	75.60	32.83				
**Zarcone et al., 1987**	8	12	91.16	20.19	99.62	22.73	22.99	4.20	4.04	0.26	53.46	14.90	49.68	15.57				
**Zarcone et al., 1995**	8	18	65.10		75.07		20.20	4.00	20.80	3.70	52.20	32.80	53.60	14.00				

Study	N. SZA	REMT (SZA)	SD	N. HC	REMT (HC)	SD
Benson et al., 1985	8	68.65	27.48	13	95.30	28.20
Benson et al., 1985	9	92.30	38.80	19	98.90	21.00
Zarcone et al., 1987	8	91.16	20.19	18	101.62	13.17
Zarcone et al., 1995	8	65.10	29.22	10	86.40	20.05
	33	79.70	29.22	60	96.85	20.05

Study	N. SZA	%REM (SZA)	SD	N. HC	%REM (HC)	SD
Benson et al., 1980	9	23.66	5.98	19	22.38	5.02
Benson et al., 1985	8	18.35	7.65	13	22.40	7.45
Benson et al., 1985	9	22.70	7.85	19	22.70	5.40
Zarcone et al., 1987	8	22.98	4.20	18	22.57	3.16
Zarcone et al., 1995	8	20.20	4.00	10	20.80	4.50
	42	21.65	5.98	79	22.30	5.02

Study	N. SZA	REML (SZA)	SD	N. HC	REML (HC)	SD
Benson et al., 1980	9	75.98	28.33	19	96.66	34.76
Benson et al., 1985	8	74.20	41.45	13	89.90	40.50
Benson et al., 1985	9	73.80	24.65	19	82.20	32.90
Zarcone et al., 1987	8	53.46	14.90	18	88.60	42.79
Zarcone et al., 1995	8	52.20	32.80	10	65.30	16.40
	42	66.35	28.33	60	86.26	34.76

Study	N. SZA	REMT (SZA)	SD	N. MDD	REMT (MDD)	SD
Kupfer & Foster, 1975	6	81.00	10.50	9	51.30	7.00
Kupfer et al., 1979	12	47.22	25.60	29	57.24	35.85
Benson et al., 1983	2	90.20	25.60	5	106.66	35.85
Benson et al., 1985	8	68.65	27.48	10	89.40	53.70
Benson et al., 1985	9	92.30	38.80	7	109.60	52.70
Zarcone et al., 1987	8	91.16	20.19	12	105.12	32.78
Zarcone et al., 1995	8	65.10	25.60	11	83.25	35.85
	53	72.89	25.60	83	78.23	35.85

Study	N. SZA	%REM (SZA)	SD	N. MDD	%REM (MDD)	SD
Kupfer & Foster, 1975	6	23.20	1.90	9	19.60	2.40
Kupfer et al., 1979	12	17.70	6.60	29	21.10	5.30
Benson et al., 1980	9	23.66	5.65	6	30.23	6.71
Benson et al., 1983	2	22.96	5.65	5	24.97	6.71
Benson et al., 1985	8	18.35	7.65	10	19.90	11.95
Benson et al., 1985	9	22.70	7.85	7	26.30	14.30
Zarcone et al., 1987	8	22.98	4.20	12	25.28	7.10
Zarcone et al., 1995	8	20.20	4.00	11	21.80	3.90
	62	21.08	5.65	89	22.71	6.71

Study	N. SZA	REML (SZA)	SD	N. MDD	REML (MDD)	SD

Study	N. SZA	REMT (SZA)	SD	N. SCZ	REMT (SCZ)	SD
Benson et al., 1983	2	90.20	25.60	7	100.37	18.53
Benson et al., 1985	8	68.65	27.48	11	98.00	40.30
Benson et al., 1985	9	92.30	38.80	8	101.70	19.45
Zarcone et al., 1987	8	91.16	20.19	12	99.62	22.73
Zarcone et al., 1995	8	65.10	25.60	18	75.07	18.53
Reich et al., 1975	3	81.00	10.50	14	54.00	5.60
Reich et al., 1975	3	81.00	10.50	9	74.80	5.60
	41	80.40	25.60	79	83.17	18.53

Study	N. SZA	%REM (SZA)	SD	N. SCZ	%REM (SCZ)	SD
Benson et al., 1980	9	23.66	5.36	9	25.44	2.86
Benson et al., 1983	2	22.96	5.36	7	26.01	2.86
Benson et al., 1985	8	18.35	7.65	11	25.85	5.70
Benson et al., 1985	9	22.70	7.85	8	24.45	4.78
Zarcone et al., 1987	8	22.99	4.20	12	4.04	0.26
Zarcone et al., 1995	8	20.20	4.00	18	20.80	3.70
Reich et al., 1975	3	23.20	1.90	14	17.40	1.60
Reich et al., 1975	3	23.20	1.90	9	20.00	1.40
	50	21.89	5.36	88	19.74	2.86

Study	N. SZA	REML (SZA)	SD	N. SCZ	REML (SCZ)	SD
Benson et al., 1980	9	75.98	24.85	9	66.20	19.65
Benson et al., 1983	2	68.25	24.85	7	50.89	19.65
Benson et al., 1985	8	74.20	41.45	11	62.90	41.00
Benson et al., 1985	9	73.80	24.65	8	75.60	32.83
Zarcone et al., 1987	8	53.46	14.90	12	49.68	15.57
Zarcone et al., 1995	8	52.20	32.80	18	53.60	14.00
Reich et al., 1975	3	31.70	5.70	14	97.90	11.00
Reich et al., 1975	3	31.70	5.70	9	81.00	12.00
	50	62.27	24.85	88	67.15	19.65

Study	N. SZA	REMD (SZA)	SD	N. SCZ	REMD (SCZ)	SD
Benson et al., 1983	2	2.62	0.20	7	2.66	0.10
Reich et al., 1975	3	2.20	0.20	14	1.60	0.10
Reich et al., 1975	3	2.20	0.20	9	1.50	0.10
	8	2.31	0.20	30	1.82	0.10

Legend
Drug-free SZA vs HC
Drug-free SZA vs MDD
Drug-free SZA vs SCZ
Calculated from original data
SD not available in original data (averaged from similar available data)

**Kupfer & Foster, 1975**	3	31.70	5.70	9	36.00	7.20
**Kupfer & Foster, 1975**	3	31.70	5.70	25	40.91	6.05
**Kupfer et al., 1979**	12	23.41	27.90	29	41.20	33.40
**Benson et al., 1980**	9	75.98	25.57	6	49.33	22.90
**Coble et al., 1981**	5	49.87	25.57	17	46.04	22.90
**Benson et al., 1983**	2	68.25	25.57	5	62.28	22.90
**Benson et al., 1985**	8	74.20	41.45	10	63.60	17.80
**Benson et al., 1985**	9	73.80	24.65	7	44.30	65.70
**Zarcone et al., 1987**	8	53.46	14.90	12	48.77	24.46
**Zarcone et al., 1995**	8	52.20	32.80	11	45.50	22.10
	67	54.39	25.57	131	45.52	22.90

**Study**	**N. SZA**	**REMD (SZA)**	**SD**	**N. MDD**	**REMD (MDD)**	**SD**
**Kupfer & Foster, 1975**	6	2.33	0.71	9	2.35	0.95
**Kupfer et al., 1979**	12	1.34	0.71	29	1.55	0.95
**Benson et al., 1983**	2	2.62	0.71	5	2.61	0.95
	20	1.77	0.71	43	1.84	0.95

Study	N. (SZA)/N. (HC)	Age (SZA/HC)	Males % (SZA/HC)	Diagnosis (SZA/HC)	Treatment
**Benson et al., 1980**	9/19	?/?	?/?	Schizoaffective disorder/Healthy	Drug-free (≥2 weeks)
**Benson et al., 1985**	8/13	33/29.7	100/100	Schizoaffective disorder/Healthy	Drug-free (≥2 weeks)
**Benson et al., 1985**	9/19	33.4/30.2	100/100	Schizoaffective disorder/Healthy	Drug-free (≥2 weeks)
**Zarcone et al., 1987**	8/18	33.8/30.3	100/100	Schizoaffective disorder/Healthy	Drug-free (≥2 weeks)
**Zarcone et al., 1995**	8/10	34.5/32.3	100/100	Schizoaffective disorder/Healthy	Drug-free (2 days)

Study	N. SZA	N. HC	ST1 (SZA)	SD	ST1 (HC)	SD	%ST1 (SZA)	SD	%ST1 (HC)	SD
**Benson et al., 1980**	9	19								
**Benson et al., 1985**	8	13								
**Benson et al., 1985**	9	19								
**Zarcone et al., 1987**	8	18								
**Zarcone et al., 1995**	8	10	50.26		80.17		15.6	5.6	19.30	6.40

Study	ST2 (SZA)	SD	ST2 (HC)	SD	%ST2 (SZA)	SD	%ST2 (HC)	SD	ST3 (SZA)	SD	ST3 (HC)	SD	%ST3 (SZA)	SD	%ST3 (HC)	SD
**Benson et al., 1980**	206.98		224.25						30.74		35.97					
**Benson et al., 1985**																
**Benson et al., 1985**																
**Zarcone et al., 1987**																
**Zarcone et al., 1995**	163.68		209.78		50.80	6.20	50.50	7.30								

Study	ST4 (SZA)	SD	ST4 (HC)	SD	%ST4 (SZA)	SD	%ST4 (HC)	SD	DELTA (SZA)	SD	DELTA (HC)	SD	%DELTA (SZA)	SD	%DELTA (HC)	SD
**Benson et al., 1980**	12.62		49.34		3.17		11.02		43.36		85.31					
**Benson et al., 1985**	3.25	14.05	46.50	44.30	0.75	4.73	10.00	10.50								
**Benson et al., 1985**	6.60	18.05	46.10	32.00	1.60	4.55	10.40	6.80								
**Zarcone et al., 1987**									42.94	32.25	83.18	30.90				
**Zarcone et al., 1995**									43.17		38.63		13.40	8.10	9.30	6.20

Study	N. (SZA)/N. (MDD)	Age (SZA/MDD)	Males % (SZA/MDD)	Diagnosis (SZA/MDD)	Treatment
**Kupfer & Foster, 1975**	6/9	38.8/58.2	50/?	Schizoaffective disorder/Psychotic depression	Drug-free (≥2 weeks)
**Kupfer & Foster, 1975**	6/25	38.8/41.1	50/?	Schizoaffective disorder/MDD	Drug-free (≥2 weeks)
**Kupfer et al., 1979**	12/29	44.4/48.1	42/31	Schizoaffective disorder/Psychotic depression	Drug-free (≥2 weeks)
**Benson et al., 1980**	9/6	?/?	?/?	Schizoaffective disorder/	Drug-free (≥2 weeks)
**Benson et al., 1985**	8/10	33/40.3	100/100	Schizoaffective disorder/MDD	Drug-free (≥2 weeks)
**Benson et al., 1985**	9/7	33.4/37.7	100/100	Schizoaffective disorder/MDD	Drug-free (≥2 weeks)
**Zarcone et al., 1987**	8/12	33.8/41.33	100/100	Schizoaffective disorder/MDD	Drug-free (≥2 weeks)
**Zarcone et al., 1995**	8/11	34.5/37.2	100/100	Schizoaffective disorder/MDD	Drug-free (2 days)

Study	N. SZA	N. MDD	ST1 (SZA)	SD	ST1 (MDD)	SD	%ST1 (SZA)	SD	%ST1 (MDD)	SD
**Kupfer & Foster, 1975**	6	9								
**Kupfer & Foster, 1975**	6	25								
**Kupfer et al., 1979**	12	29	24.55		33.37		9.20	5.30	12.30	6.40
**Benson et al., 1980**	9	6								
**Benson et al., 1985**	8	10								
**Benson et al., 1985**	9	7								
**Zarcone et al., 1987**	8	12								
**Zarcone et al., 1995**	8	11	50.26		61.49		15.6	5.6	16.1	4.7

Study	ST2 (SZA)	SD	ST2 (MDD)	SD	%ST2 (SZA)	SD	%ST2 (MDD)	SD	ST3 (SZA)	SD	ST3 (MDD)	SD	%ST3 (SZA)	SD	%ST3 (MDD)	SD
**Kupfer & Foster, 1975**																
**Kupfer & Foster, 1975**																
**Kupfer et al., 1979**	184.90		172.00		69.30	8.60	63.40	10.10								
**Benson et al., 1980**	206.98		172.88						30.74		45.11					
**Benson et al., 1985**																
**Benson et al., 1985**																
**Zarcone et al., 1987**																
**Zarcone et al., 1995**	163.68		207.37		50.80	6.20	54.3	4.5								

Study	ST4 (SZA)	SD	ST4 (MDD)	SD	%ST4 (SZA)	SD	%ST4 (MDD)	SD	DELTA (SZA)	SD	DELTA (MDD)	SD	%DELTA (SZA)	SD	%DELTA (MDD)	SD
**Kupfer & Foster, 1975**									18.20	4.50	4.40	2.20	4.80	1.20	1.60	0.85
**Kupfer & Foster, 1975**									18.20	4.50	21.00	5.70				
**Kupfer et al., 1979**									1.07		0.81		0.40	1.10	0.30	1.70
**Benson et al., 1980**	12.62		19.92		3.17		4.72		43.36		65.03					
**Benson et al., 1985**	3.25	14.05	3.70	20.70	0.75	4.73	0.90	4.75								
**Benson et al., 1985**	6.60	18.05	26.20	31.70	1.60	4.55	6.50	6.80								
**Zarcone et al., 1987**									42.94	32.25	54.18	35.92				
**Zarcone et al., 1995**									43.17		29.79		13.40	8.10	7.80	6.70

Study	N. (SZA)/N. (SCZ)	Age (SZA/SCZ)	Males % (SZA/SCZ)	Diagnosis (SZA/SCZ)	Treatment
**Reich et al., 1975**	3/14	38.8/20.8	50/57	Schizoaffective disorder/Schizophrenia (acute)	Drug-free (≥2 weeks)
**Reich et al., 1975**	3/9	38.8/19	50/11	Schizoaffective disorder/Schizophrenia (latent)	Drug-free (≥2 weeks)
**Benson et al., 1980**	9/9	?/?	?/?	Schizoaffective disorder/	Drug-free (≥2 weeks)
**Benson et al., 1985**	8/11	33/27	100/100	Schizoaffective disorder/Schizophrenia	Drug-free (≥2 weeks)
**Benson et al., 1985**	9/8	33.4/26.6	100/100	Schizoaffective disorder/Schizophrenia	Drug-free (≥2 weeks)
**Zarcone et al., 1987**	8/12	33.8/26.2	100/100	Schizoaffective disorder/Schizophrenia	Drug-free (≥2 weeks)
**Zarcone et al., 1995**	8/18	34.5/33.1	100/100	Schizoaffective disorder/Schizophrenia	Drug-free (2 days)

Study	N. SZA	N. SCZ	ST1 (SZA)	SD	ST1 (SCZ)	SD	%ST1 (SZA)	SD	%ST1 (SCZ)	SD
**Reich et al., 1975**	3	14	34.20	8.90	26.40	4.30	8.68		7.95	
**Reich et al., 1975**	3	9	34.20	8.90	29.80	5.30	8.68		8.65	
**Benson et al., 1980**	9	9								
**Benson et al., 1985**	8	11								
**Benson et al., 1985**	9	8								
**Zarcone et al., 1987**	8	12								
**Zarcone et al., 1995**	8	18	50.26		50.53		15.6	5.6	14.00	4.70

Study	ST2 (SZA)	SD	ST2 (SCZ)	SD	%ST2 (SZA)	SD	%ST2 (SCZ)	SD	ST3 (SZA)	SD	ST3 (SCZ)	SD	%ST3 (SZA)	SD	%ST3 (SCZ)	SD
**Reich et al., 1975**	217.80	18.30	194.70	11.80	62.98		63.81									
**Reich et al., 1975**	217.80	18.30	220.80	10.80	62.98		58.93									
**Benson et al., 1980**	206.98		199.24						30.74		36.71					
**Benson et al., 1985**																
**Benson et al., 1985**																
**Zarcone et al., 1987**																
**Zarcone et al., 1995**	163.68		198.13		50.80	6.20	54.90	6.30								

Study	ST4 (SZA)	SD	ST4 (SCZ)	SD	%ST4 (SZA)	SD	%ST4 (SCZ)	SD	DELTA (SZA)	SD	DELTA (SCZ)	SD	%DELTA (SZA)	SD	%DELTA (SCZ)	SD
**Reich et al., 1975**									18.20	4.50	28.90	5.70	5.26		9.47	
**Reich et al., 1975**									18.20	4.50	43.30	7.70	5.26		11.56	
**Benson et al., 1980**	12.62		21.17		3.17		5.54		43.36		57.88					
**Benson et al., 1985**	3.25	14.05	18.00	19.30	0.75	4.73	4.98	4.20								
**Benson et al., 1985**	6.60	18.05	12.25	35.40	1.60	4.55	3.30	9.78								
**Zarcone et al., 1987**									42.94	32.25	54.22	32.79				
**Zarcone et al., 1995**									43.17		36.81		13.40	8.10	10.20	7.20

Study	N. SZA	ST4 (SZA)	SD	N. HC	ST4 (HC)	SD
Benson et al., 1980	9	12.62	16.17	19	49.34	37.00
Benson et al., 1985	8	3.25	14.05	13	46.50	44.30
Benson et al., 1985	9	6.60	18.05	19	46.10	32.00
	26	7.65	16.17	51	47.41	37.00

Study	N. SZA	%ST4 (SZA)	SD	N. HC	%ST4 (HC)	SD
Benson et al., 1980	9	3.17	4.63	19	11.02	8.30
Benson et al., 1985	8	0.75	4.73	13	10.00	10.50
Benson et al., 1985	9	1.60	4.55	19	10.40	6.80
	26	1.88	4.63	51	10.53	8.30

Study	N. SZA	DELTA (SZA)	SD	N. HC	DELTA (HC)	SD
Benson et al., 1980	9	43.36	32.25	19	85.31	30.90
Zarcone et al., 1987	8	42.94	32.25	18	83.18	30.90
Zarcone et al., 1995	8	43.17	32.25	10	38.63	30.90
	25	43.16	32.25	47	74.56	30.90

Study	N. SZA	ST4 (SZA)	SD	N. MDD	ST4 (MDD)	SD
Benson et al., 1980	9	12.62	16.17	6	19.92	25.23
Benson et al., 1985	8	3.25	14.05	10	3.70	20.70
Benson et al., 1985	9	6.60	18.05	7	26.20	31.70
	26	7.65	16.17	23	14.78	25.23

Study	N. SZA	%ST4 (SZA)	SD	N. MDD	%ST4 (MDD)	SD
Benson et al., 1980	9	3.17	4.63	6	4.72	5.59
Benson et al., 1985	8	0.75	4.73	10	0.90	4.75
Benson et al., 1985	9	1.60	4.55	7	6.50	6.80
	26	1.88	4.63	23	3.60	5.59

Study	N. SZA	DELTA (SZA)	SD	N. MDD	DELTA (MDD)	SD
Kupfer & Foster, 1975	6	18.20	4.50	9	4.40	2.20
Kupfer & Foster, 1975	6	18.20	4.50	25	21.00	5.70
Kupfer et al., 1979	12	1.07	15.60	29	0.81	12.90
Benson et al., 1980	9	43.36	15.60	6	65.03	12.90
Zarcone et al., 1987	8	42.94	32.25	12	54.18	35.92
Zarcone et al., 1995	8	43.17	15.60	11	29.79	12.90
	49	26.74	15.60	92	21.26	12.90

Study	N. SZA	%DELTA (SZA)	SD	N. MDD	%DELTA (MDD)	SD
Kupfer & Foster, 1975	6	4.80	1.20	9	1.60	0.85
Kupfer et al., 1979	12	0.40	1.10	29	0.30	1.70
Zarcone et al., 1995	8	13.40	8.10	11	7.80	6.70
	26	5.42	3.28	49	2.22	2.67

Study	N. SZA	ST1 (SZA)	SD	N. SCZ	ST1 (SCZ)	SD
Reich et al., 1975	3	34.20	8.90	14	26.40	4.30
Reich et al., 1975	3	34.20	8.90	9	29.80	5.30
Zarcone et al., 1995	8	50.26	8.90	18	50.53	4.69
	14	43.38	8.90	41	37.74	4.69

Study	N. SZA	%ST1 (SZA)	SD	N. SCZ	%ST1 (SCZ)	SD
Reich et al., 1975	3	8.68	5.6	14	7.95	4.70
Reich et al., 1975	3	8.68	5.6	9	8.65	4.70
Zarcone et al., 1995	8	15.60	5.6	18	14.00	4.70
	14	12.63	5.6	41	10.76	4.70

Study	N. SZA	ST2 (SZA)	SD	N. SCZ	ST2 (SCZ)	SD
Reich et al., 1975	3	217.80	18.30	14	194.70	11.80
Reich et al., 1975	3	217.80	18.30	9	220.80	10.80
Benson et al., 1980	9	206.98	18.30	9	199.24	11.41
Zarcone et al., 1995	8	163.68	18.30	18	198.13	11.41
	23	194.74	18.30	50	201.45	11.41

Study	N. SZA	%ST2 (SZA)	SD	N. SCZ	%ST2 (SCZ)	SD
Reich et al., 1975	3	62.98	6.20	14	63.81	6.30
Reich et al., 1975	3	62.98	6.20	9	58.93	6.30
Zarcone et al., 1995	8	50.80	6.20	18	54.90	6.30
	14	56.02	6.20	41	58.83	6.30

Study	N. SZA	ST4 (SZA)	SD	N. SCZ	ST4 (SCZ)	SD
Benson et al., 1980	9	12.62	16.17	9	21.17	26.08
Benson et al., 1985	8	3.25	14.05	11	18.00	19.30
Benson et al., 1985	9	6.60	18.05	8	12.25	35.40
	26	7.65	16.17	28	17.38	26.08

Study	N. SZA	%ST4 (SZA)	SD	N. SCZ	%ST4 (SCZ)	SD
Benson et al., 1980	9	3.17	4.63	9	5.54	6.55
Benson et al., 1985	8	0.75	4.73	11	4.98	4.20
Benson et al., 1985	9	1.60	4.55	8	3.30	9.78
	26	1.88	4.63	28	4.68	6.55

Study	N. SZA	DELTA (SZA)	SD	N. SCZ	DELTA (SCZ)	SD
Reich et al., 1975	3	18.20	4.50	14	28.90	5.70
Reich et al., 1975	3	18.20	4.50	9	43.30	7.70
Benson et al., 1980	9	43.36	20.36	9	57.88	15.50
Zarcone et al., 1987	8	42.94	32.25	12	54.22	32.79
Zarcone et al., 1995	8	43.17	20.36	18	36.81	15.50
	31	38.33	20.36	62	42.39	15.50

Drug-free SZA vs HC
Drug-free SZA vs MDD
Drug-free SZA vs SCZ
Calculated from original data
SD not available in original data (averaged from similar available data)

Study	N. SZA	%DELTA (SZA)	SD	N. SCZ	%DELTA (SCZ)	SD
**Reich et al., 1975**	3	5.26	8.10	14	9.47	7.20
**Reich et al., 1975**	3	5.26	8.10	9	11.56	7.20
**Zarcone et al., 1995**	8	13.40	8.10	18	10.20	7.20
	14	9.91	8.10	41	10.25	7.20

Study	N. (SZA)/N. (HC)	Age (SZA/HC)	Males % (SZA/HC)	Diagnosis (SZA/HC)	Treatment
**Benson et al., 1980**	9/19	?/?	?/?	Schizoaffective disorder/Healthy	Drug-free (≥2 weeks)
**Benson et al., 1985**	8/13	33/29.7	100/100	Schizoaffective disorder/Healthy	Drug-free (≥2 weeks)
**Benson et al., 1985**	9/19	33.4/30.2	100/100	Schizoaffective disorder/Healthy	Drug-free (≥2 weeks)
**Zarcone et al., 1987**	8/18	33.8/30.3	100/100	Schizoaffective disorder/Healthy	Drug-free (≥2 weeks)
**Zarcone et al., 1995**	8/10	34.5/32.3	100/100	Schizoaffective disorder/Healthy	Drug-free (2 days)

Study	N. SZA	N. HC	TST (SZA)	SD	TST (HC)	SD	SOL (SZA)	SD	SOL (HC)	SD	SE (SZA)	SD	SE (HC)	SD	WASO (SZA)	SD	WASO (HC)	SD
**Benson et al., 1980**	9	19					36.77		9.88						41.62		8.43	10.94
**Benson et al., 1985**	8	13	354.25	111.20	441.40	44.05	47.5	27.88	6.2	5.40								
**Benson et al., 1985**	9	19	409.10	44.70	451.80	23.50	42.70	30.00	8.50	6.60								
**Zarcone et al., 1987**	8	18	394.89	36.98	451.15	17.78	60.12	32.07	16.08	10.59					44.45	38.67	7.72	6.08
**Zarcone et al., 1995**	8	10	322.20	101.40	415.40	38.40	74.80	69.10	15.00	9.90	70.90	22.20	90.70	5.40	53.00	46.00	30.40	19.70

Study	N. (SZA)/N. (MDD)	Age (SZA/MDD)	Males % (SZA/MDD)	Diagnosis (SZA/MDD)	Treatment
**Kupfer & Foster, 1975**	6/9	38.8/58.2	50/?	Schizoaffective disorder/Psychotic depression	Drug-free (≥2 weeks)
**Kupfer et al., 1979**	12/29	44.4/48.1	42/31	Schizoaffective disorder/Psychotic depression	Drug-free (≥2 weeks)
**Benson et al., 1980**	9/6	?/?	?/?	Schizoaffective disorder/	Drug-free (≥2 weeks)
**Benson et al., 1983**	2/5	?/?	100/100	Schizoaffective disorder/MDD	Drug-free (≥2 weeks)
**Benson et al., 1985**	8/10	33/40.3	100/100	Schizoaffective disorder/MDD	Drug-free (≥2 weeks)
**Benson et al., 1985**	9/7	33.4/37.7	100/100	Schizoaffective disorder/MDD	Drug-free (≥2 weeks)
**Zarcone et al., 1987**	8/12	33.8/41.33	100/100	Schizoaffective disorder/MDD	Drug-free (≥2 weeks)
**Zarcone et al., 1995**	8/11	34.5/37.2	100/100	Schizoaffective disorder/MDD	Drug-free (2 days)

Study	N. SZA	N. MDD	TST (SZA)	SD	TST (MDD)	SD	SOL (SZA)	SD	SOL (MDD)	SD	SE (SZA)	SD	SE (MDD)	SD	WASO (SZA)	SD	WASO (MDD)	SD
**Kupfer & Foster, 1975**	6	9	345.80	28.40	262.90	23.70					73.00	4.00	61.00	5.00	14.70	5.70	54.50	13.40
**Kupfer et al., 1979**	12	29	266.80	73.40	271.30	79.00	79.00	54.50	63.30	41.90	64.70	16.60	62.40	22.80	40.10	47.50	47.80	41.90
**Benson et al., 1980**	9	6					36.77		32.78						41.62		19.33	
**Benson et al., 1983**	2	5	392.90		427.22		57.70		24.36									
**Benson et al., 1985**	8	10	354.25	111.20	426.10	43.40	47.5	27.88	12.00	19.40								
**Benson et al., 1985**	9	7	409.10	44.70	403.80	40.40	42.70	30.00	8.90	52.60								
**Zarcone et al., 1987**	8	12	394.89	36.98	410.74	36.99	60.12	32.07	34.69	31.41					44.45	38.67	32.26	36.98
**Zarcone et al., 1995**	8	11	322.20	101.40	381.90	81.50	74.80	69.10	23.80	16.20	70.90	22.20	84.90	14.40	53.00	46.00	42.00	43.30

Study	N. (SZA)/N. (SCZ)	Age (SZA/SCZ)	Males % (SZA/SCZ)	Diagnosis (SZA/SCZ)	Treatment
**Reich et al., 1975**	3/14	38.8/20.8	50/57	Schizoaffective disorder/Schizophrenia (acute)	Drug-free (≥2 weeks)
**Reich et al., 1975**	3/9	38.8/19	50/11	Schizoaffective disorder/Schizophrenia (latent)	Drug-free (≥2 weeks)
**Benson et al., 1980**	9/9	?/?	?/?	Schizoaffective disorder/Schizophrenia	Drug-free (≥2 weeks)
**Benson et al., 1983**	2/7	?/?	100/100	Schizoaffective disorder/Schizophrenia	Drug-free (≥2 weeks)
**Benson et al., 1985**	8/11	33/27	100/100	Schizoaffective disorder/Schizophrenia	Drug-free (≥2 weeks)
**Benson et al., 1985**	9/8	33.4/26.6	100/100	Schizoaffective disorder/Schizophrenia	Drug-free (≥2 weeks)
**Zarcone et al., 1987**	8/12	33.8/26.2	100/100	Schizoaffective disorder/Schizophrenia	Drug-free (≥2 weeks)
**Zarcone et al., 1995**	8/18	34.5/33.1	100/100	Schizoaffective disorder/Schizophrenia	Drug-free (2 days)

Study	N. SZA	N. SCZ	TST (SZA)	SD	TST (SCZ)	SD	SOL (SZA)	SD	SOL (SCZ)	SD	SE (SZA)	SD	SE (SCZ)	SD	WASO (SZA)	SD	WASO (SCZ)	SD
**Reich et al., 1975**	3	14	345.80	28.40	305.10	16.40	102.20	17.70	79.60	18.40	73.00	4.00	72.00	4.00	14.70	5.70	30.10	10.00
**Reich et al., 1975**	3	9	345.80	28.40	374.70	11.40	102.20	17.70	43.40	5.70	73.00	4.00	87.00	2.00	14.70	5.70	6.20	3.40
**Benson et al., 1980**	9	9					36.77		38.22						41.62		12.64	20.77
**Benson et al., 1983**	2	7	392.90		385.87		57.70		67.10									
**Benson et al., 1985**	8	11	354.25	111.20	413.30	87.60	47.5	27.88	33.20	63.00								
**Benson et al., 1985**	9	8	409.10	44.70	399.95	61.37	42.70	30.00	47.15	23.83								
**Zarcone et al., 1987**	8	12	394.89	36.98	385.77	50.50	60.12	32.07	63.99	30.58					44.45	38.67	16.26	9.77
**Zarcone et al., 1995**	8	18	322.20	101.40	360.90	58.10	74.80	69.10	67.40	60.50	70.90	22.20	79.60	12.90	53.00	46.00	29.20	28.10

Study	N. SZA	TST (SZA)	SD	N. HC	TST (HC)	SD
Benson et al., 1985	8	354.25	111.20	13	441.40	44.05
Benson et al., 1985	9	409.10	44.70	19	451.80	23.50
Zarcone et al., 1987	8	394.89	36.98	18	451.15	17.78
Zarcone et al., 1995	8	322.20	101.40	10	415.40	38.40
	33	371.29	72.70	60	443.29	28.72

Study	N. SZA	SOL (SZA)	SD	N. HC	SOL (HC)	SD
Benson et al., 1980	9	36.77	39.47	19	9.88	5.40
Benson et al., 1985	8	47.50	27.88	13	6.2	5.40
Benson et al., 1985	9	42.70	30.00	19	8.50	6.60
Zarcone et al., 1987	8	60.12	32.07	18	16.08	10.59
Zarcone et al., 1995	8	74.80	69.10	10	15.00	9.90
	42	51.78	39.47	79	11.00	8.09

Study	N. SZA	WASO (SZA)	SD	N. HC	WASO (HC)	SD
Benson et al., 1980	9	41.62	42.34	19	8.43	10.94
Zarcone et al., 1987	8	44.45	38.67	18	7.72	6.08
Zarcone et al., 1995	8	53.00	46.00	10	30.40	19.70
	25	46.17	42.34	47	12.83	10.94

Study	N. SZA	TST (SZA)	SD	N. MDD	TST (MDD)	SD
Kupfer & Foster, 1975	6	345.80	28.40	9	262.90	23.70
Kupfer et al., 1979	12	266.80	73.40	29	271.30	79.00
Benson et al., 1983	2	392.90	67.65	5	427.22	58.48
Benson et al., 1985	8	354.25	111.20	10	426.10	43.40
Benson et al., 1985	9	409.10	44.70	7	403.80	40.40
Zarcone et al., 1987	8	394.89	36.98	12	410.74	36.99
Zarcone et al., 1995	8	322.20	101.40	11	381.90	81.50
	53	345.56	67.65	83	344.43	58.48

Study	N. SZA	SOL (SZA)	SD	N. MDD	SOL (MDD)	SD
Kupfer et al., 1979	12	79.00	54.50	29	63.30	41.90
Benson et al., 1980	9	36.77	43.48	6	32.78	33.80
Benson et al., 1983	2	57.70	43.48	5	24.36	33.80
Benson et al., 1985	8	47.50	27.88	10	12.00	19.40
Benson et al., 1985	9	42.70	30.00	7	8.90	52.60
Zarcone et al., 1987	8	60.12	32.07	12	34.69	31.41
Zarcone et al., 1995	8	74.80	69.10	11	23.80	16.20
	56	57.82	43.48	80	37.68	33.80

Study	N. SZA	WASO (SZA)	SD	N. MDD	WASO (MDD)	SD
Kupfer & Foster, 1975	6	14.70	5.70	9	54.50	13.40
Kupfer et al., 1979	12	40.10	47.50	29	47.80	41.90
Benson et al., 1980	9	41.62	37.69	6	19.33	36.98
Zarcone et al., 1987	8	44.45	38.67	12	32.26	36.98

Study	N. SZA	TST (SZA)	SD	N. SCZ	TST (SCZ)	SD
Benson et al., 1983	2	392.90	65.88	7	385.87	48.07
Benson et al., 1985	8	354.25	111.20	11	413.30	87.60
Benson et al., 1985	9	409.10	44.70	8	399.95	61.37
Zarcone et al., 1987	8	394.89	36.98	12	385.77	50.50
Zarcone et al., 1995	8	322.20	101.40	18	360.90	58.10
Reich et al., 1975	3	345.80	28.40	14	305.10	16.40
Reich et al., 1975	3	345.80	28.40	9	374.70	11.40
	41	368.62	65.88	79	369.82	48.07

Study	N. SZA	SOL (SZA)	SD	N. SCZ	SOL (SCZ)	SD
Reich et al., 1975	3	102.20	17.70	14	79.60	18.40
Reich et al., 1975	3	102.20	17.70	9	43.40	5.70
Benson et al., 1980	9	36.77	36.12	9	38.22	36.78
Benson et al., 1983	2	57.70	36.12	7	67.10	36.78
Benson et al., 1985	8	47.50	27.88	11	33.20	63.00
Benson et al., 1985	9	42.70	30.00	8	47.15	23.83
Zarcone et al., 1987	8	60.12	32.07	12	63.99	30.58
Zarcone et al., 1995	8	74.80	69.10	18	67.40	60.50
	44	58.06	36.12	65	57.30	36.78

Study	N. SZA	WASO (SZA)	SD	N. SCZ	WASO (SCZ)	SD
Benson et al., 1980	9	41.62	32.34	9	12.64	14.97
Zarcone et al., 1987	8	44.45	38.67	12	16.26	9.77
Zarcone et al., 1995	8	53.00	46.00	18	29.20	28.10
Reich et al., 1975	3	14.70	5.70	14	30.10	10.00
Reich et al., 1975	3	14.70	5.70	9	6.20	3.40
	31	40.08	32.34	62	21.16	14.97

Study	N. SZA	SE (SZA)	SD	N. SCZ	SE (SCZ)	SD
Reich et al., 1975	3	73.00	4.00	14	72.00	4.00
Reich et al., 1975	3	73.00	4.00	9	87.00	2.00
Zarcone et al., 1995	8	70.90	22.20	18	79.60	12.90
	14	71.80	14.40	41	78.63	7.47

Drug-free SZA vs HC
Drug-free SZA vs MDD
Drug-free SZA vs SCZ
Calculated from original data
SD not available in original data (averaged from similar available data)

**Zarcone et al., 1995**	8	53.00	46.00	11	42.00	43.30
	43	40.08	37.69	67	42.41	36.98

Study	N. SZA	SE (SZA)	SD	N. MDD	SE (MDD)	SD
**Kupfer & Foster, 1975**	6	73.00	4.00	9	61.00	5.00
**Kupfer et al., 1979**	12	64.70	16.60	29	62.40	22.80
**Zarcone et al., 1995**	8	70.90	22.20	11	84.90	14.40
	26	68.52	15.42	49	67.19	17.64

Study (excluded)	N. (SZA)/N. (CTRL)	Age (SZA/CTRL)	Males % (SZA/CTRL)	Diagnosis (SZA/CTRL)	Treatment	Reason of exclusion
**Kupfer et al., 1971**	4	36.48	25	1 Schizophrenia<br>**1 Schizoaffective disorder**<br>2 Depressive neurosis	Drug-free (≥2 weeks) →<br>Chlorpromazine	Mixed sample
**Gillin et al., 1977**	10	25	60	**4 Schizoaffective disorder**<br>2 Acute schizophrenia<br>1 Chronic schizophrenia<br>1 Schizoid personality disorder<br>2 Bipolar disorder	Drug-free (≥2 weeks) →<br>Pimozide	Mixed sample
**Gillin et al., 1978**	7	23	43	3 Chronic schizophrenia<br>1 Acute schizophrenia<br>**2 Schizoaffective disorder**<br>1 Schizoid personality disorder + OCD	Drug-free (≥1 month) →<br>Pimozide	Mixed sample
**Keshavan et al., 1996**	16	29.9	50	11 Schizophrenia<br>**5 Schizoaffective disorder**	Drug-free (≥2 weeks) →<br>**Drug-free (31%)**<br>Neuroleptics (69%)<br>+ Antidepressants<br>+ Mood stabilizers	Mixed medication status/sample
**Keshavan et al., 1996**	15	29.7	?	Schizophrenia<br>**5 Schizoaffective disorder**	Drug-free (≥2 weeks) -><br>Haloperidol (67%)<br>Fluphenazine (13%)<br>Trifluoperazine (6.7%)<br>Clozapine (6.7%)<br>Risperidone (6.7%)<br>+ Anticholinergics (47%)	Mixed sample
**Benson et al., 1996**	14/15	33.6/30.5	100/100	11 Schizophrenia (presumably chronic)<br>**3 Schizoaffective disorder**/Healthy	Drug-free (2 days)	Mixed sample
**Nishino et al., 1998**	14/14	33.3/34.4	100/100	12 Schizophrenia<br>**2 Schizoaffective disorder**/Healthy	Drug-free (2 days)	Mixed sample
**Keshavan et al., 1998**	30/30	30.9/30.9	57/57	19 Schizophrenia<br>**8 Schizoaffective disorder**<br>3 Schizophreniform disorder/Healthy	Drug-free (Average 97.5 weeks;<br>Median 40 weeks)	Mixed sample
**Yamashita et al., 1999**	1	34	0	**Schizoaffective disorder**<br>(with haloperidol-induced akathisia)	Haloperidol + Chlorpromazine<br>+ Biperiden + BZD<br>+ Mood stabilizer<br>→ BZD + Mood stabilizer	Single-case study
**Rotenberg et al., 2000**	20/10	45.8/43.3	65/60	19 Schizophrenia<br>**1 Schizoaffective disorder**/Healthy	Drug-free (≥2 weeks)	Mixed sample
**Wetter et al., 2002**	1	31	0	**Schizoaffective disorder**<br>(with risperidone-induced<br>restless leg syndrome)	Risperidone<br>+ Mood stabilizers<br>→ Quetiapine<br>+ Mood stabilizers	Single-case study
**Armitage et al., 2004**	14	40.9	29	**3 Schizoaffective disorder**<br>11 Bipolar I disorder	Carbamazepine, BZD,<br>mood stabilizers<br>→ + Clozapine	Mixed sample
**Kluge et al., 2014**	15	32.8	47	Schizophrenia<br>**1 Schizoaffective disorder**	Drug-free (≥2 days) -><br>Olanzapine	Mixed sample
**Kluge et al., 2014**	15	36.7	33	Schizophrenia<br>**2 Schizoaffective disorder**<br>1 Schizophreniform disorder	Drug-free (≥2 days) -><br>Clozapine	Mixed sample
**Göder et al., 2015**	16/16	29.4/28.3	56/44	14 Chronic schizophrenia<br>**2 Schizoaffective disorder**/Healthy	Antipsychotics (2° Gen., 94%)<br>Antipsychotics (1° Gen., 6%)<br>+ Antidepressants (13%)<br>+ BZD (6%)<br>+ Anticholinergics (6%)<br>+ Promethazine (6%)	Mixed sample
**Kaskie et al., 2019**	27/23	23.2/24.7	68/72	16 Schizophrenia<br>**2 Schizoaffective disorder**<br>4 Unspecified psychosis<br>3 MDD with psychotic features<br>2 Bipolar disorder/Healthy	Antipsychotics (2° Gen., 41%)<br>Antipsychotics (3° Gen., 3.7%)<br>+ Antidepressants (83%)<br>+ Mood stabilizers (25%)<br>+ BZD (17%)<br><br>**15 drug-naive**	Mixed medication status/sample
**Gerstenberg et al., 2020**	12/24	16.6/16.5	58/46	9 Schizophrenia<br>**2 Schizoaffective Disorder**<br>1 Brief Psychotic Disorder/Healthy	Antipsychotics (3° Gen., 58.3%)<br>Antipsychotics (2°+3° Gen., 25.5%)<br>Antipsychotics (2° Gen., 16.2%)<br>+ BZD (25%)<br>+ Anticholinergics (16.7%)	Mixed sample
**Petit et al., 2022**	12/988	58.2/59.9	42/58	**Schizoaffective disorder**/Healthy	Medicated (25%)<br>**Drug-free (75%)**	Mixed medication status
**Denis et al., 2024**	17/27	21/24	56/53	7 Schizophrenia<br>**5 Schizoaffective disorder**<br>4 Bipolar disorder<br>1 Unspecified psychosis/Healthy	Antipsychotics (2° Gen., 35%)<br>Antipsychotics (3° Gen., 29%)<br>Antipsychotics (1° Gen., 12%)<br>+ Mood stabilizers (47%)<br>+ Antidepressants (24%)<br>+ Anticholinergics (18%)<br>+ BZD (6%)<br><br>**4 drug-free**	Mixed medication status/sample

Study (excluded)	N. SZA	N. CTRL	TST (SZA)	SD	TST (CTRL)	SD	LAT (SZA)	SD	LAT (CTRL)	SD	SE (SZA)	SD	SE (CTRL)	SD	WASO (SZA)	SD	WASO (CTRL)	SD
**Kupfer et al., 1971**	4	4	311.30	63.90	354.30	48.30	57.30	25.00	53.90	24.90					54.80	37.90	21.10	17.40
**Gillin et al., 1977**	10	10	352.00	46.00	404.00	52.00					80.70	7.30	87.30	7.50	14.00	21.00	11.00	8.00
**Gillin et al., 1978**	7	7	334.00	18.00	385.00	12.00					82.70	5.10	84.50	1.90				
**Keshavan et al., 1996**	16	16	325.00	92.70	436.80	77.30	63.80	62.50	53.40	36.70	72.17		83.61		61.50	84.00	32.20	19.30
**Keshavan et al., 1996**	15	15	340.40	101.90	404.40	48.60	49.60	39.50	21.70	12.20	74.73		90.01		65.60	85.50	17.80	15.50
**Benson et al., 1996**	14	15	334.20	56.00	446.50	17.80	92.40	71.50	15.70	11.10	74.80	14.20	94.60	2.60	28.00	26.30	7.20	6.70
**Nishino et al., 1998**	14	14	365.90	57.80	425.80	28.30	71.60	62.80	17.30	12.40	78.90	13.40	91.40	4.10	30.00	25.80	25.90	17.60
**Keshavan et al., 1998**	30	30	347.60	69.80	429.40	44.00	51.80	52.10	10.60	7.10	77.90	13.90	94.40	3.30	49.30	43.20	15.00	13.60
**Yamashita et al., 1999**	1	1									86.80		84.40					
**Rotenberg et al., 2000**	20	10	413.00	74.00	412.00	43.00	32.00	69.00	3.30	2.10	92.00	11.00	98.00	3.00				
**Wetter et al., 2002**	1	1	412.50		470.50		71.50		14.50		85.30		96.60		71.00		13.00	
**Armitage et al., 2004**	14	14	406.30	74.50	495.80	94.70	20.90	23.30	44.20	30.90	92.50	6.40	91.90	4.50	24.78		32.72	
**Kluge et al., 2014**	15	15	350.90	75.40	459.60	24.80	42.20	30.20	19.40	20.40	83.00	14.00	97.00	1.00				
**Kluge et al., 2014**	15	15	354.40	90.70	457.20	24.00	29.90	26.60	14.00	18.00	82.00	13.00	97.00	3.00				
**Göder et al., 2015**	16	16	428.70	72.90	369.70	42.20	34.60	35.30	21.00	17.60	85.80	8.90	86.60	7.80				
**Kaskie et al., 2019**	27	23	389.00	88.00	428.00	50.00	43.00	53.00	15.00	12.00					77.00	59.00	59.00	34.00
**Gerstenberg et al., 2020**	12	24					24.10	16.60	16.10	9.00	91.50	5.50	91.70	5.00	22.60	20.40	25.00	17.80
**Petit et al., 2022**	12	988	386.40	78.60	394.80	69.00	31.00		16.00		78.80		83.90		107.00		78.00	
**Dan et al., 2024**	17	27	507.00	54.00	508.00	74.00	20.00	20.00	25.00	29.00	85.00	9.00	86.00	8.00	65.00	49.00	52.00	41.00

Study (excluded)	REMT (SZA)	SD	REMT (CTRL)	SD	%REM (SZA)	SD	%REM (CTRL)	SD	REML (SZA)	SD	REML (CTRL)	SD	REMD (SZA)	SD	REMD (CTRL)	SD
**Kupfer et al., 1971**	59.80	23.90	65.30	17.00	19.10	3.80	18.50	4.10	57.90	22.60	60.30	41.10	1.61		1.66	
**Gillin et al., 1977**	82.00	15.00	95.00	16.00	22.80	3.50	24.30	3.20	64.00	18.00	65.00	35.00	1.70	0.40	1.50	0.50
**Gillin et al., 1978**	80.00	6.00	92.00	7.00	23.00	1.60	21.10	1.80	46.00	13.00	64.00	13.00	1.80	0.20	1.80	0.10
**Keshavan et al., 1996**	76.20	35.30	98.90	35.30	22.30	8.30	22.20	5.60	61.20	24.40	84.60	44.20	1.29	0.45	1.57	0.44
**Keshavan et al., 1996**	77.60	39.00	91.90	23.70	21.60	8.00	22.70	4.90	56.00	26.50	76.20	36.80	1.40	0.70	1.50	0.80
**Benson et al., 1996**	63.83		103.59		19.10	3.20	23.20	3.20	62.60	21.10	94.40	44.70				
**Nishino et al., 1998**	75.70	22.90	93.20	27.40	20.70	4.40	22.00	5.80	65.30	21.90	73.20	13.30				
**Keshavan et al., 1998**	78.60	27.40	107.60	25.70	22.60	6.30	24.90	5.10	60.90	27.00	69.10	30.30	1.50	0.60	1.40	0.50
**Yamashita et al., 1999**					17.80		20.60									
**Rotenberg et al., 2000**	93.75		79.52		22.70	8.00	19.30	3.70	93.40	6.10	89.40	33.70	5.10	3.55	4.07	2.54
**Wetter et al., 2002**	70.00		66.50		16.97		14.13									
**Armitage et al., 2004**	51.60		62.97		12.70	5.70	12.70	5.20	115.90	41.90	118.70	49.20	2.50	1.10	2.90	0.80
**Kluge et al., 2014**	77.70	24.20	115.20	25.50	22.14		25.06		116.40	57.60	88.00	55.90	2.62	1.53	3.25	1.05
**Kluge et al., 2014**	77.90	34.70	99.90	44.50	21.98		21.85		94.00	40.50	105.00	48.00	3.11	0.84	3.07	1.45
**Göder et al., 2015**	87.88		68.76		20.50	5.10	18.60	4.60					14.10	6.70	11.00	4.00
**Kaskie et al., 2019**	81.69		81.32		21.00	7.00	19.00	6.00								
**Gerstenberg et al., 2020**									152.20	67.40	122.50	45.20				
**Petit et al., 2022**	85.00		87.00		21.70		21.80		114.00		87.00					
**Dan et al., 2024**	93.00	42.00	113.00	33.00	18.10	7.60	21.90	5.10								

Study (excluded)	ST1 (SZA)	SD	ST1 (CTRL)	SD	%ST1 (SZA)	SD	%ST1 (CTRL)	SD	ST2 (SZA)	SD	ST2 (CTRL)	SD	%ST2 (SZA)	SD	%ST2 (CTRL)	SD
**Kupfer et al., 1971**																
**Gillin et al., 1977**	33.00	51.00	29.00	18.00	5.30	3.30	7.20	4.40	216.00	25.00	255.00	32.00	59.90	5.00	63.80	6.00
**Gillin et al., 1978**	15.00	3.00	30.00	7.00	4.30	1.10	7.60	5.20	228.00	14.00	265.00	23.00	63.30	2.80	65.80	3.70
**Keshavan et al., 1996**																
**Keshavan et al., 1996**																
**Benson et al., 1996**	48.79		27.24		14.60	7.00	6.10	2.40	179.13		229.05		53.60	7.80	51.30	5.40
**Nishino et al., 1998**	50.90	23.40	60.10	20.60	13.90	5.40	14.60	5.30	183.50	37.40	231.10	37.40	51.30	7.00	55.30	8.60
**Keshavan et al., 1998**																
**Yamashita et al., 1999**					13.20		9.90						59.40		50.40	
**Rotenberg et al., 2000**																
**Wetter et al., 2002**	39.50		56.00						231.50		202.00					
**Armitage et al., 2004**	78.01		61.98		19.20	10.20	12.50	6.70	246.62		322.27		60.70	12.90	65.00	9.90
**Kluge et al., 2014**	35.20	20.90	31.50	19.10	10.03		6.85		183.40	60.70	206.70	40.70	52.26		44.97	
**Kluge et al., 2014**	29.60	15.10	20.10	12.40	8.35		4.40		205.10	45.20	296.60	49.30	57.87		64.87	
**Göder et al., 2015**	51.44		42.52		12.00	8.30	11.50	4.20	222.07		184.48		51.80	8.40	49.90	7.60
**Kaskie et al., 2019**	31.12		34.24		8.00	6.00	8.00	4.00	206.17		231.12		53.00	8.00	54.00	6.00
**Gerstenberg et al., 2020**																
**Petit et al., 2022**	47.00		47.00		12.40		12.20		160.00		185.00		41.60		46.90	
**Dan et al., 2024**	34.00	40.00	31.00	23.00	6.90	8.20	6.10	4.70	259.00	57.00	262.00	54.00	23.80	8.60	20.50	6.50

Study (excluded)	ST3 (SZA)	SD	ST3 (CTRL)	SD	%ST3 (SZA)	SD	%ST3 (CTRL)	SD	ST4 (SZA)	SD	ST4 (CTRL)	SD	%ST4 (SZA)	SD	%ST4 (CTRL)	SD	DELTA (SZA)	SD	DELTA (CTRL)	SD	%DELTA (SZA)	SD	%DELTA (CTRL)	SD
**Kupfer et al., 1971**																					9.20	9.70	10.10	12.00
**Gillin et al., 1977**																	38.00	21.00	21.00	16.00	10.90	5.50	6.70	5.50
**Gillin et al., 1978**																	32.00	9.00	13.00	4.00	9.70	2.30	3.50	1.20
**Keshavan et al., 1996**																	48.40	26.60	52.20	35.40	16.90	13.00	12.80	8.70
**Keshavan et al., 1996**																	44.00	29.90	56.20	37.10	14.20	11.50	13.90	8.90
**Benson et al., 1996**	39.44		37.51		11.80	6.70	8.40	4.20	3.01		49.56		0.90	1.70	11.10	4.70								
**Nishino et al., 1998**																	49.00	23.90	34.20	30.00	14.10	8.20	8.10	6.90
**Keshavan et al., 1998**																	35.00	30.10	53.10	27.40	9.90	8.30	12.20	5.90
**Yamashita et al., 1999**					0.70		5.10						0.00		0.00						0.70		5.10	
**Rotenberg et al., 2000**																	67.32		59.74		16.30	10.10	14.50	6.80
**Wetter et al., 2002**																	67.50		143.00		16.37		30.39	
**Armitage et al., 2004**																	5.28		15.37		1.30	2.30	3.10	5.30
**Kluge et al., 2014**	21.90	15.50	47.00	22.70	6.24		10.23		32.30	30.50	50.10	34.60	9.20		10.90		54.20	39.70	95.80	47.60	15.45		20.84	
**Kluge et al., 2014**	24.30	22.60	22.60	10.40	6.86		4.94		16.60	23.10	15.80	18.50	4.68		3.46		41.00	39.30	38.40	37.00	11.57		8.40	
**Göder et al., 2015**																	67.73		73.94		15.80	9.50	20.00	7.20
**Kaskie et al., 2019**																	66.13		77.04		17.00	9.00	18.00	7.00
**Gerstenberg et al., 2020**																								
**Petit et al., 2022**																	94.00		75.00		24.20		19.10	
**Dan et al., 2024**																	121.00	47.00	102.00	31.00	23.80	8.60	20.50	6.50

Study	N. SZA/Sex/(Age)	Diagnosis	Medication status	TST	SD	LAT	SD	SE	SD	WASO	SD	REMT	SD	%REM	SD	REML	SD	REMD	SD	ST1	SD	%ST1	SD	ST2	SD	%ST2	SD	ST3	SD	%ST3	SD	ST4	SD	%ST4	SD	DELTA	SD	%DELTA	SD
**Hauri et al., 1973**	2F (28.5)	Schizoaffective disorder	Drug-free (≥1 week)	411.50		8.00						113.57		27.60						24.69		6.00		231.47		56.25		14.20		3.45		0.00		0.00					
**Gillin et al., 1981**	3M, 3F (31)	4 Chronic schizophrenia<br>**2 Schizoaffective disorder**	Drug-free (≥1 week)	357.80	78.90	62.00	43.30	80.00	11.80			112.30	20.60	32.30	5.10	76.90	32.30	2.50	0.50	18.00	11.30	5.30	3.20	178.80	70.60	48.00	12.50									48.00	25.30	14.50	8.80
**Glassman et al., 1986**	1M (39)	Schizoaffective disorder	Lithium carbonate, chlorpromazine, triazolam, benztropine<br>(medication-induced somnambulism with REM suppression)	508.00								24.00		4.72		480.00				4.00		0.79		390.00		76.80		10.00		1.97		80.00		15.75		90.00		17.72	
**Lahmeyer et al., 1988**	2F (29)	Schizoaffective disorder	Drug-free (2 weeks)													80.13		1.32																					
**Keshavan et al., 1991**	7M, 5F (31.2)	8 Schizophrenia<br>**3 Schizoaffective disorder**<br>1 Schizotypal disorder	Drug-free (≥2 weeks)<br>2 drug-naive													65.10	46.50																					16.00	8.70
**Keshavan et al., 1992**	14M, 5F (30.5)	17 Schizophrenia<br>**2 Schizoaffective disorder**	Drug-free (≥2 weeks)<br>6 drug-naive													65.00	32.40																						
**Douglass et al., 1993**	6? (?)	**Schizoaffective disorder**<br>Schizophrenia	Drug-free (≥2 weeks)	230.00	78.00	92.70	58.00	64.40	22.00	30.50	52.00	58.60	20.00	22.60	6.50	41.60	102.00	1.40	0.75	87.63		38.10	25.00	108.10		47.00	12.00												
**Douglass et al., 1993**	3? (?)	Schizophrenia<br>**Schizoaffective disorder**<br>+ **narcolepsy**	Drug-free (≥2 weeks)	274.00		27.30		71.70		81.30		36.30		13.00		124.70		1.50		140.56		51.30		97.27		35.50													
**Keshavan et al., 1994**	8M, 11F (30.6)	13 Schizophrenia<br>**6 Schizoaffective disorder**<br>(suicidal behavior)	Drug-free (≥2 weeks)									96.00	24.55			54.82	27.54	1.69	0.70																				
**Keshavan et al., 1994**	12M, 8F (32.3)	16 Schizophrenia<br>**4 Schizoaffective disorder**<br>(no suicidal behavior)	Drug-free (≥2 weeks)									75.05	26.70			58.10	28.32	1.50	0.47																				
**Keshaven et al., 1995**	10M, 10F (30)	**8 Schizoaffective disorder**<br>12 Schizophrenia	Drug-free (≥4 weeks)<br>12 drug-naive	315.00	97.00			70.29																												46.90	41.70	16.70	16.20
**Baandrup et al., 2016**	5M, 5F (49)	6 Schizophrenia<br>**2 Schizoaffective disorder**<br>2 Bipolar disorder	Antipsychotics<br>+ BZD<br>+ Antidepressants<br>+ Mood stabilizers<br>+ Anticholinergics	469.30	124.60			84.90				74.15		15.80	5.00	163.80	65.50			55.38		11.80	7.50	313.96		66.90	8.30	25.81		5.50	5.90								
**Weinhold et al., 2022**	9M, 9F (40.7)	11 Schizophrenia<br>**7 Schizoaffective disorder**	Atypical antipsychotics<br>+ Antidepressants<br>+ Mood stabilizers	442.00	13.00	32.00	5.00	86.30	1.80	67.00	36.00	68.00	5.00	15.38		130.00	15.00			49.00	5.00	11.09		176.00	11.00	39.82		149.00	11.00	33.71									
			**Study summary (no clear sleep data)**																																				
**Giedke et al., 1992**	12M, 18F (48)	**4 Schizoaffective disorder**<br>26 MDD	All patients on neuroleptics, antidepressants and BZD<br>Sleep deprivation significantly improved depressed mood																																				
**Keshavan et al., 1992**	11M, 5F (31.65)	10 Schizophrenia<br>**4 Schizoaffective disorder**<br>1 Atypical psychosis<br>1 Psychotic affective disorder	Reduced NREM sleep<br>REM activity increased, REML reduced (drug-free ≥2 days)<br>Neuroleptic treatment did not improve sleep																																				

	Meta-analysis					Heterogeneity			Publication Bias			Funnel plot asymmetry		
**SZA vs HC**	**N. of studies**	**N. of subjects**	**Mean ± SD**	**RE Model**	**p-value**	**Q**	**I^2**	**p-value**	**Fail-Safe N (p-value)**	**Outlier**	**Overly influential**	**Rank correlation (p-value)**	**Regression test (p-value)**	**Power analysis**
**TST**	4	33 vs 60	371.29 ± 72.7 vs 443.29 ± 28.72	-1.43 (-1.903 to -0.949)	**0.001**	2.797	0%	p=0.424	48 (p<0.001)	-	-	p=0.7500	p=0.4260	0.99
**SOL**	5	42 vs 79	51.78 ± 39.47 vs 11 ± 8.09	1.70 (1.236 to 2.159)	**0.001**	4.379	10.43%	p=0.357	105 (p<0.001)	-	Benson et al., 1980	p=0.0833	p=0.1477	1.00
**WASO**	3	25 vs 47	46.17 ± 42.34 vs 12.83 ± 10.94	1.20 (0.649 to 1.746)	**0.001**	2.262	6.62%	p=0.323	19 (p<0.001)	-	-	p=1.0000	p=0.8293	0.98
**REMT**	4	33 vs 60	79.7 ± 29.22 vs 96.85 ± 20.05	-0.620 (-1.058 to -0.181)	**0.006**	1.495	0%	p=0.683	8 (p=0.002)	-	-	p=0.3333	p=0.2678	0.90
**%REM**	5	42 vs 79	21.65 ± 5.98 vs 22.3 ± 5.02	-0.038 (-0416 to 0.339)	0.843	1.722	0%	p=0.787	0 (p=0.399)	-	-	p=0.4833	p=0.3592	0.04
**REML**	5	42 vs 60	66.35 ± 28.33 vs 86.26 ± 34.76	-0.528 (-0.911 to -0.145)	**0.007**	1.369	0%	p=0.849	9 (p=0.003)	-	-	p=0.8167	p=0.8123	0.93
**ST4**	3	26 vs 51	7.65 ± 16.17 vs 47.41 ± 37.00	-1.20 (-1.713 to -0.693)	**0.001**	0.166	0%	p=0.920	21 (p<0.001)	-	-	p=1.0000	p=0.9619	0.99
**%ST4**	3	26 vs 51	1.88 ± 4.63 vs 10.53 ± 8.30	-1.14 (-1.649 to -0.636)	**0.001**	0.429	0%	p=0.807	19 (p<0.001)	-	-	p=1.0000	p=0.9100	0.98
**DELTA**	3	25 vs 47	43.16 ± 32.25 vs 74.56 ± 30.90	-0.814 (-1.725 to 0.097)	0.080	6.107	67.66%	p=0.047	8 (p<0.001)	Zarcone et al., 1995	-	p=0.3333	p=0.0874	0.57

	Meta-analysis					Heterogeneity			Publication Bias			Funnel plot asymmetry		
**SZA vs MDD**	**N. of studies**	**N. of subjects**	**Mean ± SD**	**RE Model**	**p-value**	**Q**	**I^2**	**p-value**	**Fail-Safe N (p-value)**	**Outlier**	**Overly influential**	**Rank correlation (p-value)**	**Regression test (p-value)**	**Power analysis**
**TST**	7	53 vs 83	345.56 ± 67.65 vs 344.43 ± 58.48	0.028 (-0.827 to 0.884)	0.948	21.237	80.04%	p=0.002	0 (p=0.431)	Kupfer & Foster, 1975	Kupfer & Foster, 1975	p=0.7726	p=0.1983	0.10
**SOL**	7	56 vs 80	57.82 ± 43.48 vs 37.68 ± 33.80	0.684 (0.321 to 1.047)	**0.001**	4.931	0%	p=0.553	31 (p<0.001)	-	-	p=1.0000	p=0.4002	1.00
**SE**	3	26 vs 49	68.52 ± 15.42 vs 67.19 ± 17.64	0.535 (-1.247 to 2.316)	0.556	14.418	90.44%	p<0.001	0 (p=0.093)	Kupfer & Foster, 1975	-	p=1.0000	p=0.2274	0.25
**WASO**	5	43 vs 67	40.08 ± 37.69 vs 42.41 ± 36.98	-0.385 (-1.649 to 0.879)	0.550	19.199	88.48%	p<0.001	0 (p=0.137)	Kupfer & Foster, 1975	Kupfer & Foster, 1975	p=1.0000	**p=0.0239***	0.23
**REMT**	7	53 vs 83	72.89 ± 25.6 vs 78.23 ± 35.85	0.009 (-0.861 to 0.879)	0.984	20.393	80.67%	p=0.002	0 (p=0.349)	Kupfer & Foster, 1975	Kupfer & Foster, 1975	p=0.2389	p=0.0652	0.10
**%REM**	8	62 vs 89	21.08 ± 5.65 vs 22.71 ± 6.71	-0.246 (-0.689 to 0.198)	0.277	11.842	37.83%	p=0.106	0 (p=0.092)	Kupfer & Foster, 1975	-	p=0.2751	p=0.3701	0.58
**REML**	10	67 vs 131	54.39 ± 25.57 vs 45.52 ± 22.90	0.033 (-0.367 to 0.434)	0.870	14.28	35.00%	p=0.113	0 (p=0.439)	-	-	p=1.000	p=0.8657	0.08
**REMD**	3	20 vs 43	1.77 ± 0.71 vs 1.84 ± 0.95	-0.150 (-0.684 to 0.384)	0.582	0.152	0%	p=0.927	0 (p=0.342)	-	-	p=1.000	p=0.7320	0.07
**ST4**	3	26 vs 23	7.65 ± 16.17 vs 14.78 ± 25.23	-0.348 (-0.922 to 0.225)	0.234	1.051	0%	p=0.591	0 (p=0.110)	-	-	p=1.000	p=0.4381	0.27
**%ST4**	3	26 vs 23	1.88 ± 4.63 vs 3.60 ± 5.59	-0.357 (-0.932 to 0.217)	0.223	1.281	0%	p=0.527	0 (p=0.104)	-	-	p=1.000	p=0.4131	0.28
**DELTA**	6	49 vs 92	26.74 ± 15.60 vs 21.26 ± 12.90	0.351 (-0.991 to 1.693)	0.609	30.314	91.28%	p<0.001	0 (p=0.184)	Kupfer & Foster, 1975	-	p=0.7194	**p=0.0379***	0.22
**%DELTA**	3	26 vs 49	5.42 ± 3.28 vs 2.22 ± 2.67	1.16 (-0.501 to 2.821)	0.171	12.554	88.30%	p=0.002	9 (p<0.001)	Kupfer & Foster, 1975	-	p=0.3333	**p=0.0005***	0.45

	Meta-analysis					Heterogeneity			Publication Bias			Funnel plot asymmetry		
**SZA vs SCZ**	**N. of studies**	**N. of subjects**	**Mean ± SD**	**RE Model**	**p-value**	**Q**	**I^2**	**p-value**	**Fail-Safe N (p-value)**	**Outlier**	**Overly influential**	**Rank correlation (p-value)**	**Regression test (p-value)**	**Power analysis**
**TST**	7	41 vs 79	368.62 ± 65.88 vs 369.82 ± 48.07	-0.052 (-0.770 to 0.666)	0.887	15.949	66.73%	p=0.014	0 (p=0.378)	Reich et al., 1975.1	-	p=1.0000	p=0.6722	0.10
**SOL**	6	44 vs 65	58.06 ± 36.12 vs 57.30 ± 36.78	0.210 (-0.160 to 0.581)	0.266	20.661	0%	p=0.004	5 (p=0.018)	Reich et al., 1975.2	-	p=0.3988	**p=0.0003***	0.34
**SE**	3	14 vs 41	71.80 + 14.40 vs 78.63 ± 7.47	-1.63 (-4.714 to 1.458)	0.301	14.999	93.61%	p<0.001	7 (p=0.002)	Reich et al., 1975.2	-	p=1.0000	**p=0.0343***	0.32
**WASO**	5	31 vs 62	40.08 ± 32.34 vs 21.16 ± 14.97	0.664 (-0.375 to 1.703)	0.210	14.377	77.93%	p=0.006	10 (p=0.003)	Reich et al., 1975.1	-	p=0.4833	p=0.8658	0.56
**REMT**	7	41 vs 79	80.4 ± 25.6 vs 83.17 ±18.53	0.218 (-0.845 to 1.281)	0.688	24.28	84.39%	p<0.001	0 (p=0.382)	Reich et al., 1975.1	Reich et al., 1975.1	p=0.0690	**p=0.0005***	0.18
**%REM**	8	50 vs 88	21.89 ± 5.36 vs 19.74 ± 2.86	1.06 (-0.724 vs 2.850)	0.244	61.892	94.47%	p<0.001	13 (p=0.005)	Zarcone et al., 1987	Zarcone et al., 1987	p=0.1789	**p<0.0001***	0.62
**REML**	8	50 vs 88	62.27 ± 24.85 vs 67.15 ± 19.65	-0.894 (.2.461 to 0.672)	0.263	41.256	93.60%	p<0.001	7 (p=0.014)	Reich et al., 1975.1	Reich et al., 1975.1	p=0.3988	**p<0.0001***	0.51
**REMD**	3	8 vs 30	2.31 ± 0.20 vs 1.82 ± 0.10	3.12 (−0.388 to 6.635)	0.081	21.365	89.27%	p<0.001	23 (p<0.001)	Benson et al., 1983	-	p=1.0000	p=0.0185	0.48
**ST1**	3	14 vs 41	43.48 ± 8.9 ± 37.74 ± 4.69	0.558 (-0.326 to 1.441)	0.216	3.517	44.12%	p=0.172	1 (p=0.043)	-	-	p=0.3333	p=0.0898	0.31
**%ST1**	3	14 vs 41	12.63 ± 5.6 vs 10.76 ± 4.70	0.203 (-0.411 to 0.817)	0.516	0.161	0%	p=0.923	0 (p=0.289)	-	-	p=0.3333	p=0.7012	0.08
**ST2**	4	23 vs 50	194.74 ± 18.30 vs 201.45 ± 11.41	-0.137 (-1.836 to 1.561)	0.874	26.218	88.52%	p<0.001	0 (p=0.250)	Zarcone et al., 1995	-	p=0.7500	p=0.5116	0.15
**%ST2**	3	14 vs 41	56.02 ± 6.20 vs 58.83 ± 6.30	-0.198 (-0.907 to 0.510)	0.583	2.372	19.39%	p=0.305	0 (p=0.327)	-	-	p=0.3333	p=0.1563	0.09
**ST4**	3	26 vs 28	7.65 ± 16.17 vs 17.38 ± 26.08	-0.461 (-1.007 to 0.084)	0.097	0.862	0%	p=0.650	1 (p=0.049)	-	-	p=1.0000	p=0.9210	0.43
**%ST4**	3	26 vs 28	1.88 ± 4.63 vs 4.68 ± 6.55	-0.507 (-1.054 to 0.040)	0.069	1.101	0%	p=0.577	1 (p=0.034)	-	-	p=1.0000	p=0.7572	0.50
**DELTA**	5	31 vs 62	38.33 ± 20.36 vs 42.39 ± 15.50	-0.984 (-2.093 to 0.126)	0.082	16.348	80.35%	p=0.003	16 (p<0.001)	-	-	**p=0.0167***	**p=0.0001***	0.73
**%DELTA**	3	14 vs 41	9.91 ± 8.19 ± 10.25 ± 7.20	-0.167 (-0.972 to 0.638)	0.685	2.916	35.41%	p=0.233	0 (p=0.276)	-	-	p=0.3333	p=0.0878	0.10

*Asymmetry found

Meta-analysis studies	Case Definition Adequacy	Representativeness of the Cases	Selection of Controls	Definition of Controls	Comparability	Ascertainment of Exposure	Same method of Ascertainment	Non-Response Rate	Total NOS Score
Benson et al., 1980	1	1	1	1	0	1	1	1	7
Benson et al., 1985	1	1	1	1	1	1	1	1	8
Zarcone et al., 1987	1	0	1	1	1	1	1	0	6
Zarcone et al., 1995	1	0	1	1	1	1	1	0	6
Kupfer & Foster, 1975	1	1	1	1	0	1	1	1	7
Kupfer et al., 1979	1	1	1	1	2	1	1	1	9
Coble et al., 1981	1	1	1	1	1	1	1	1	8
Benson et al., 1983	1	1	1	1	1	1	1	0	7
Reich et al., 1975	1	1	1	1	1	1	1	1	8
**Case-control studies, excluded**									
Benson et al., 1996	1	0	0	0	1	1	1	0	4
Nishino et al., 1998	1	0	1	1	2	1	1	1	8
Keshavan et al., 1998	1	0	1	1	1	1	1	0	6
Rotenberg et al., 2000	1	0	1	1	1	1	1	0	6
Göder et al., 2015	1	1	1	1	1	1	1	1	8
Kaskie et al., 2019	1	1	1	1	2	1	1	1	9
Gerstenberg et al., 2020	1	0	1	1	1	1	1	0	6
Petit et al., 2022	1	1	1	1	2	1	1	1	9
Denis et al., 2024	1	1	1	1	2	1	1	0	8
